# Dig up tall fescue plastid genomes for the identification of morphotype-specific DNA variants

**DOI:** 10.1186/s12864-023-09631-8

**Published:** 2023-10-03

**Authors:** Md. Shofiqul Islam, Konstantin Chekhovskiy, Malay C. Saha

**Affiliations:** 1 Grass Genomics, Noble Research Institute LLC, 2510 Sam Noble Parkway, Ardmore, OK 73401 USA; 2 Genetics Laboratory, Indiana Crop Improvement Association, 7700 Stockwell Road, Lafayette, IN 47909 USA; 3 Department of Agronomy, Purdue University, 915 Mitch Daniels Blvd, West Lafayette, IN 47906 USA

**Keywords:** Chloroplast genome, *Festuca*, Genomic divergence, InDels, Morphotype, SNPs, Tall fescue

## Abstract

**Background:**

Tall fescue (*Festuca arundinacea* Schreb.) is an important cool-season perennial grass species. Hexaploid tall fescue has three distinct morphotypes used either as forage or turf purposes. Its chloroplast genome is conserved due to it being maternally inherited to the next generation progenies. To identify morphotype-specific DNA markers and the genetic variations, plastid genomes of all three tall fescue morphotypes, i.e., Continental cv. Texoma MaxQ II, Rhizomatous cv. Torpedo, and Mediterranean cv. Resolute, have been sequenced using Illumina MiSeq sequencing platform.

**Results:**

The plastid genomes of Continental-, Rhizomatous-, and Mediterranean tall fescue were assembled into circular master molecules of 135,283 bp, 135,336 bp, and 135,324 bp, respectively. The tall fescue plastid genome of all morphotypes contained 77 protein-coding, 20 tRNAs, four rRNAs, two pseudo protein-coding, and three hypothetical protein-coding genes. We identified 630 SNPs and 124 InDels between Continental and Mediterranean, 62 SNPs and 20 InDels between Continental and Rhizomatous, and 635 SNPs and 123 InDels between Rhizomatous and Mediterranean tall fescue. Only four InDels in four genes (*ccsA*, *rps18*, *accD*, and *ndhH-p*) were identified, which discriminated Continental and Rhizomatous plastid genomes from the Mediterranean plastid genome. Here, we identified and reported eight InDel markers (NRITCHL18, NRITCHL35, NRITCHL43, NRITCHL65, NRITCHL72, NRITCHL101, NRITCHL104, and NRITCHL110) from the intergenic regions that can successfully discriminate tall fescue morphotypes. Divergence time estimation revealed that Mediterranean tall fescue evolved approximately 7.09 Mya, whereas the divergence between Continental- and Rhizomatous tall fescue occurred about 0.6 Mya.

**Conclusions:**

To our knowledge, this is the first report of the assembled plastid genomes of Rhizomatous and Mediterranean tall fescue. Our results will help to identify tall fescue morphotypes at the time of pre-breeding and will contribute to the development of lawn and forage types of commercial varieties.

**Supplementary Information:**

The online version contains supplementary material available at 10.1186/s12864-023-09631-8.

## Background

Tall fescue (*Festuca arundinacea* Schreb.) is an outbreeding allohexaploid (2n = 6x = 42) member of the Poaceae grass family and is used as pasture and turf grass. As a member of a polyploid species, genetic variation exists within allohexaploid tall fescue results in three distinct morphotypes such as Continental, Rhizomatous, and Mediterranean [1]. The Continental morphotype originated from northern Europe, and is highly productive as it provides high quality forage and is active during summer months, but persistence is badly hampered during extreme hot and dry summer months when cultivated at the edge of their adaptability in the southern Great Plains [2, 3]. The Mediterranean morphotype originated from the Mediterranean Basin of southern Europe and northern Africa persists well under extreme drought and heat stresses and exhibits incomplete summer dormancy. This is an important drought avoidance phenomenon [4]. Summer dormancy of Mediterranean tall fescue has been correlated with plant survival, fall regrowth, and persistence under severe drought as compared to the Continental type. Therefore, integration of summer dormancy in Continental tall fescue is of great interest to the forage and turf grass breeder since drought and heat stresses are the most likely to increase due to global warming and may affect plant persistence [5]. The Rhizomatous morphotype can be found at the northern part of Portugal and at Galicia in Spain [6]. It produces longer rhizomes that are absent in Continental and Mediterranean morphotypes [7]. This trait is the choice of turf breeding program due to its superior spreading ability and preventing soil erosion [8].

It is a tedious task to distinguish Continental-, Rhizomatous-, and Mediterranean tall fescue morphotypes with a visual inspection on the above ground morphological traits unless the Rhizomatous morphotype develops rhizome in their root system and the Mediterranean morphotype expresses dormancy during hot summer months. On the other hand, DNA sequence variation is a powerful tool that can be used to separate tall fescue morphotype from each other within a quick turnaround time. Though the advent of the high-throughput next-generation sequencing technologies has changed the landscape of genomics and transcriptomics research, the nuclear DNA sequences of tall fescue is not available to date (October 31, 2022) due to a heterozygous genetic background [9]. In addition, chloroplast genome has gained considerable sequence variation within and between plant species [10]. Thereby, sequencing of the chloroplast genome has the potential to increase our understanding of the climatic adaptation of economically important tall fescue morphotypes.

Chloroplasts are semi-autonomous membrane-bound cytoplasmic organelles known as plastids that conduct photosynthesis in green plants. They have their own genome of 120–160 kb in length with quadripartite structure found in most of the green plants [11]. The plastid genome contains genes for ribosomal ribonucleic acids (rRNAs), transfer ribonucleic acids (tRNAs), and subunits of the photosystem. Therefore, to conserve DNA sequence in a fairly small genome and maternal inheritance, the plastid genome has become a molecular tool to investigate the genetic inheritance and phylogenetic relationships among species [12–14]. The first plastid genome of tobacco (*Nicotiana tabacum*) was released in 1986 [15]. It is desirable to generate completely assembled plastid genomes to facilitate the molecular study of species speciation. In tall fescue, more than a decade ago, a plastid genome of Continental tall fescue cv. Kentucky 31 (KY31) was sequenced and assembled [12]. However, the complete plastid genome sequence of Rhizomatous and Mediterranean tall fescue is hitherto unknown. In this study we sequenced and assembled chloroplast genomes of the Rhizomatous and Mediterranean morphotypes and re-sequenced Continental morphotype cv. Texoma MaxQ II to identify DNA polymorphisms such as: single nucleotide polymorphisms (SNPs), insertions and deletions (InDels), simple sequence repeats (SSRs), and large inverted repeats (IRs) among them.

The specific objectives are to: (1) assemble the complete plastid genomes of the three tall fescue morphotypes, (2) identify DNA polymorphisms among the plastid genomes, (3) to develop morphotype-specific InDel markers, and (4) to estimate the genomic divergence among tall fescue morphotypes. The outcome of this study will improve our knowledge of genetic diversity and variability among the three tall fescue morphotypes. The complete plastid genome sequences of tall fescue morphotypes will then be used to develop tall fescue genotype/variety-specific molecular markers, which will facilitate the breeding of tall fescue for the identification and conservation of valuable agronomic traits.

## Results

### Sequencing and assembly of the tall fescue plastid genome

A total of 13,458,542 paired-end sequence reads were generated using Illumina MiSeq system with an average read length of 300 bp from plastid genomes of the three tall fescue morphotypes (Table 1). A total of 9,357,442 reads survived after removing Illumina adapter, low-quality reads and contaminating mitochondrial reads. The clean reads were assembled by performing a reference-guided assembly which resulted in 7 (both in Continental and Rhizomatous) and 8 (Mediterranean morphotype) gaps ranging from 0.2 to 2.1 Kb (Table 1). Later, these gaps were closed via PCR amplification followed by Sanger sequencing and finally, circular master molecules were generated for each of the three morphotypes.

**Table 1:** Summary of tall fescue plastid genome sequence reads and reference-guided assembly

Tall fescue morphotypes^*^	No. of Illumina paired end reads	No. of reads used in genome assembly^§^	No. of gaps	Gap length (Kb)	Gaps closed by PCR
Continental	4,760,498	3,346,272	7	0.30-2.0	Yes
Rhizomatous	4,474,942	3,033,103	7	0.30–1.9	Yes
Mediterranean	4,223,102	2,978,067	8	0.20–2.1	Yes
Total	13,458,542	9,357,442	22	-	-

^*^Continental cv. Texoma MaxQ II, Rhizomatous cv. Torpedo, and Mediterranean cv. Resolute

^§^Reads were quality trimmed and removed mitochondrial reads using a reference perennial ryegrass mitochondrial genome (GenBank Accession no. JX999996.1)

### Features of plastid genomes of tall fescue morphotypes

**Fig. 1:**
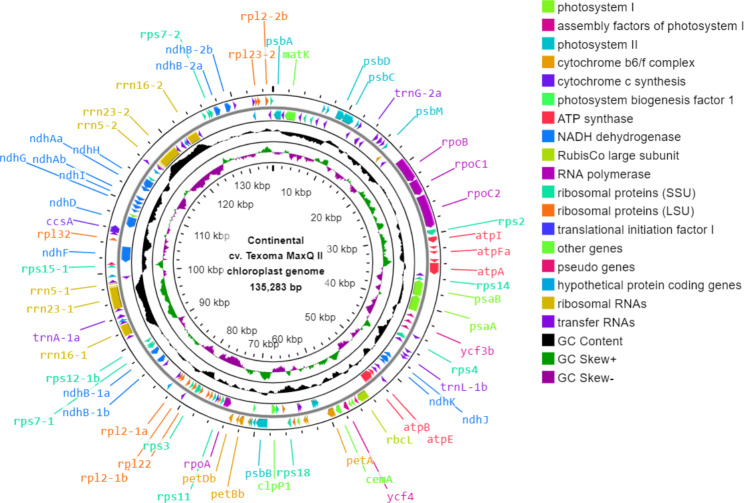
**Map of Continental cv. Texoma MaxQ II tall fescue plastid genome**. The genes encoding proteins, tRNAs, and rRNAs were shown using the arrowhead on the in- and outside of the main circle. The second outer circle represents the master molecule. The genes located in the forward and reverse strands were visualized in the clockwise and anticlockwise orientation, respectively. The middle and innermost circles represent the GC content and GC skew in the plastid genome, respectively. Some of the gene names were displayed in the figure and can be found in Additional file 1: Table S1

**Fig. 2:**
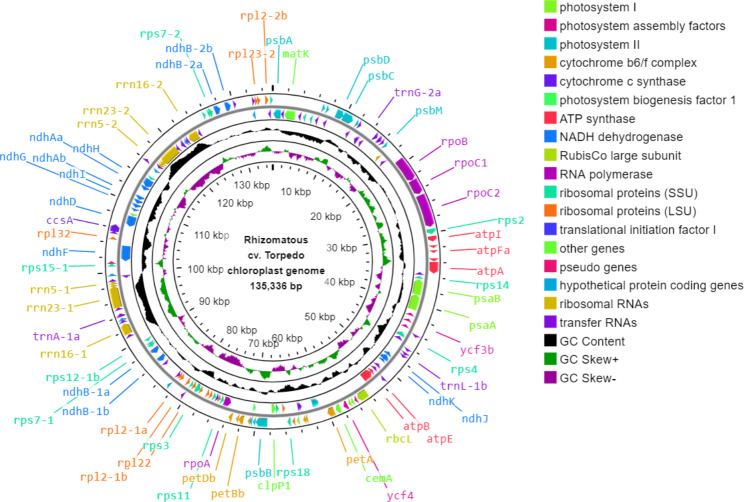
**Map of Rhizomatous cv. Torpedo tall fescue plastid genome**. The genes encoding proteins, tRNAs, and rRNAs were shown using the arrowhead on the in- and outside of the main circle. The second outer circle represents the master molecule. The genes located in the forward and reverse strands were visualized in the clockwise and anticlockwise orientation, respectively. The middle and innermost circles represent the GC content and GC skew in the plastid genome, respectively. Some of the gene names were displayed in the figure and can be found in Additional file 1: Table S2

**Fig. 3:**
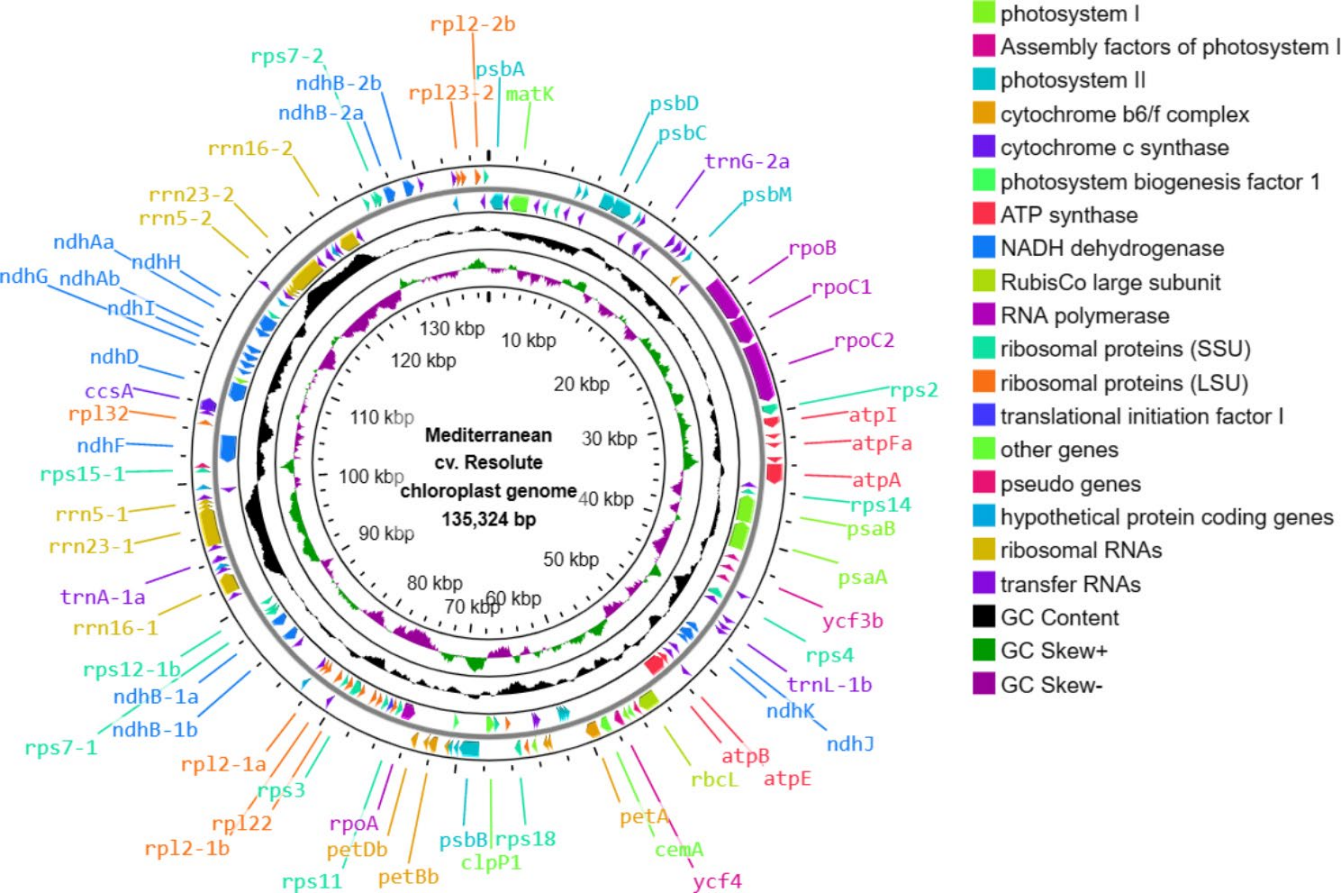
**Map of Mediterranean cv. Resolute tall fescue plastid genome**. The genes encoding proteins, tRNAs, and rRNAs were shown using the arrowhead on the in- and outside of the main circle. The second outer circle represents the master molecule. The genes located in the forward and reverse strands were visualized in the clockwise and anticlockwise orientation, respectively. The middle and innermost circles represent the GC content and GC skew in the plastid genome, respectively. Some of the gene names were displayed in the figure and can be found in Additional file 1: Table S3

The plastid genome size of tall fescue morphotypes were 135,283 bp for Continental, 135,336 bp for Rhizomatous, and 135,324 bp for Mediterranean with GC content ranged from 38.2 to 38.3% (Figs. 1, 2 and 3; Table 2).

**Table 2:** Features of plastid genomes of Continental-, Rhizomatous-, and Mediterranean tall fescue morphotypes

	Continental cv. Texoma MaxQ II	Rhizomatous cv. Torpedo	Mediterranean cv. Resolute
Genome size (bp)	135,283	135,336	135,324
-Length of large single copy (LSC) region (bp)	79,953	80,008	79,950
-Length of inverted repeat (IR) region (bp)	42,840^a^	42,840^a^	42,881^a^
-Length of small single copy (SSC) region (bp)	12,490	12,488	12,493
GC content (%)	38.3	38.2	38.3
Gene number			
-Protein-coding genes -Pseudo protein-coding genes -Hypothetical protein-coding genes -rRNA genes -tRNA genes	84(77)^b^ 2 6(3)^b^ 8(4)^b^ 41(20)^b^	84(77)^b^ 2 6(3)^b^ 8(4)^b^ 41(20)^b^	84(77)^b^ 2 6(3)^b^ 8(4)^b^ 41(20)^b^
^**^Proteins			
-Protein coding sequences (bp) -Protein coding sequence (%)	60,813 44.95	60,813 44.93	60,861 44.97
RNAs			
-rRNA gene coding sequences (bp) -rRNA gene coding sequence (%) -tRNA gene coding sequences (bp) -tRNA gene coding sequence (%)	9,194 6.80 3,068 2.27	9,194 6.79 3,068 2.27	9,194 6.79 3,068 2.27
^***^Introns			
-Cis-spliced group II intron -Trans-spliced group II intron -Intron sequences of protein and RNA-coding genes (bp) -Intron sequence of protein and RNA-coding genes (%)	14 2 15,585 11.52	14 2 15,624 11.54	14 2 15,571 11.51

^a^sum of a pair of inverted repeats length; ^b^non-redundant; bp, base pairs

^**^Including pseudo protein-coding genes and hypothetical protein-coding genes

^***^The length of the *matK* and *ycf68* genes were excluded from the intron sequences of *trnK* and *trnI* genes, respectively, as they were located within the intron sequences. In addition, trans-spliced intron sequences (between *rps12-1a* and *rps12-1b*, and between *rps12-2a* and *rps12-2b*) were not included

The plastid genome of tall fescue morphotypes contains 84 genes encoding 77 different proteins, two pseudo protein-coding genes (*ndhH*-p and *rpl*23-p), and three hypothetical protein-coding genes (*ycf*1, *ycf*2, and *ycf*68) present in two copies each (Figs. 1, 2 and 3; Table 2, Additional file 1: Table S1, Table S2, and Table S3). Among the protein-coding genes, seven genes (*ndhB, rps*7, *rps*12, *rps*15, *rps*19, *rp*l2, *and rpl*23) had two copies each. In total, the protein-coding genes (including pseudo and hypothetical protein-coding genes) cover 44.95%, 44.93% and 44.97% of plastid genomes of Continental, Rhizomatous, and Mediterranean tall fescue, respectively (Table 2).

The plastid genome of tall fescue morphotypes contains four rRNA genes (present in two copies each) for the ribosomal subunits 4.5 S, 5 S, 16 S, and 23 S (Figs. 1, 2 and 3). The length of the rRNA genes ranged from 95 to 2,889 nucleotides and represented 6.80% of the plastid genome (Table 2, Additional file 1: Table S1, Table S2, and Table S3). The plastid genome of each of the tall fescue morphotype contains a total of 41 tRNA-coding genes matching the codons of 20 amino acids (Figs. 1, 2 and 3). Among the tRNA genes, *trnA*, *trnG*, *trnH*, *trnI*, *trnL*, *trnM*, *trnN*, *trnR*, *trnS*, *trnT*, and *trnV* had at least two copies each. The length of the tRNA genes ranged from 59 to 88 nucleotides and covered 2.27% of the plastid genome (Table 2, Additional file 1: Table S1, Table S2, and Table S3). Among the 84 protein-coding genes, only 13 (*ycf3*, *petB*, *petD*, *atpF*, *ndhA*, *ndhB*-1, *ndhB*-2, *rps12*-1, *rps12*-2, *rps16*, *rpl2*-1, *rpl2*-2, and *rpl16*) contain introns. Sixteen group II introns (two trans-spliced introns that belongs to *rps12*-1 and *rps12*-2, and 14 cis-spliced) were found within the 13 protein-coding genes. Among the 41 tRNA genes, eight (*trnA*-1, *trnA*-2, *trnG*-2, *trnI*-2, *trnI*-3, *trnK*, *trnL*-1, and *trnV*-1) contain introns. None of the four rRNA genes contain any intron (Additional file 1: Table S1, Table S2, and Table S3).

### Expansion and contraction of the LSC, IRs, and SSC border regions

**Fig. 4:**
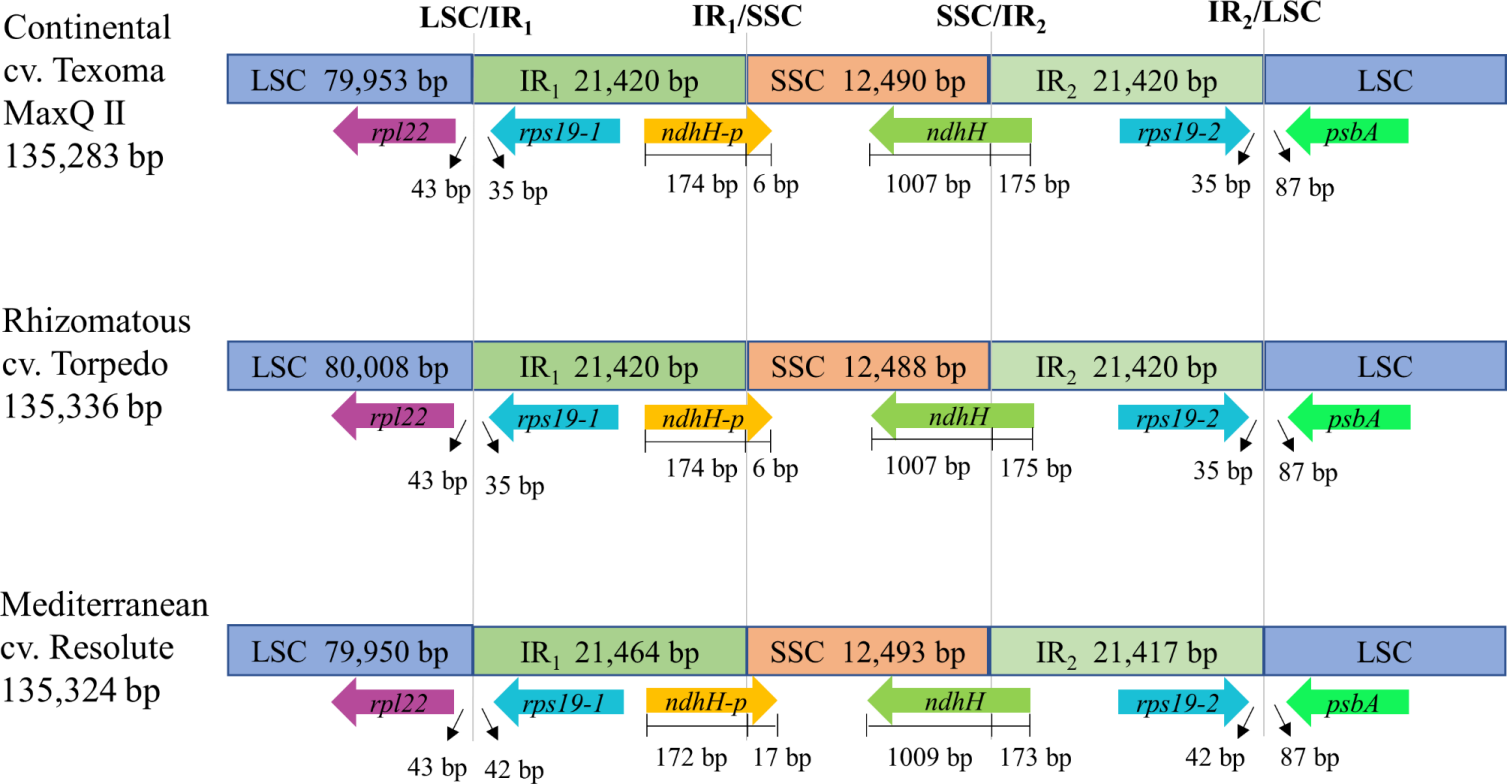
**The comparison of LSC, IRs, and SSC border regions in the three tall fescue morphotype plastid genomes**. The adjacent border genes were shown in forward and reverse strand using the right and left block arrow, respectively. The figure is not scaled according to the sequence length and showed the distance of the adjacent genes at or near the SC/IR borders

The plastid genome of tall fescue showed quadripartite structure consisting of a large single copy (LSC) and a small single copy (SSC) separated from LSC by a pair of large IR regions (IR1 and IR2). The physical boundaries of these four regions are presented in Fig. 4. The largest LSC identified in Rhizomatous (80,008 bp) while the smallest LSC region identified in Mediterranean (79,950 bp) plastid genome. The length of SSC varied from 12,488 bp (Rhizomatous) to 12,493 bp (Mediterranean) (Fig. 4; Table 2). The plastid genomes of Continental and Rhizomatous morphotypes contained equal length of two IRs (IR1: 21,420 bp and IR2: 21,420 bp), but interestingly, there was a 47-bp deletion between the two IRs (IR1: 21,464 bp and IR2: 21,417 bp) in Mediterranean morphotype (Fig. 4).

The boundaries of the border region and adjacent genes of the three plastid genomes were analyzed to understand the expansion and contraction variation in the adjacent regions. Results suggest that the junction positions of LSC/IR1, IR1/SSC, SSC/IR2, and IR2/LSC were not conserved in these plastid genomes. For example, the *rpl22* gene, which was located 43 bp away from the LSC/IR1 boundary in the LSC region in all the three plastid genomes. On the other hand, the *rps19-1* gene located 35 bp away from the LSC/IR1 boundary in the IR1 region of the Continental and Rhizomatous plastid genomes, but 42 bp away from the LSC/IR1 region of the Mediterranean plastid genome, leading to a complete duplication of the gene *rps19-2* in the IR2 region. In Continental and Rhizomatous plastid genomes, *ndhH* gene was located in the SSC/IR2 region with a length of 1182 bp, including 1007 bp in the SSC and 175 bp in the IR2, leading to an incomplete duplication of the gene and formation of a pseudo gene, *ndhH-p* (180 bp) within the IR1/SSC region. However, in the Mediterranean plastid genome, the *ndhH* gene showed similar length of 1182 bp that covers 1009 bp in the SSC and 173 bp in the IR2 region, leading to an incomplete duplication of the gene and formation of a pseudo gene, *ndhH-p* (189 bp) within the IR1/SSC region. The Mediterranean *ndhH-p* gene was 9 bp longer than that of Continental and Rhizomatous (Fig. 4).

### Repeat features in the plastid genome

**Fig. 5:**
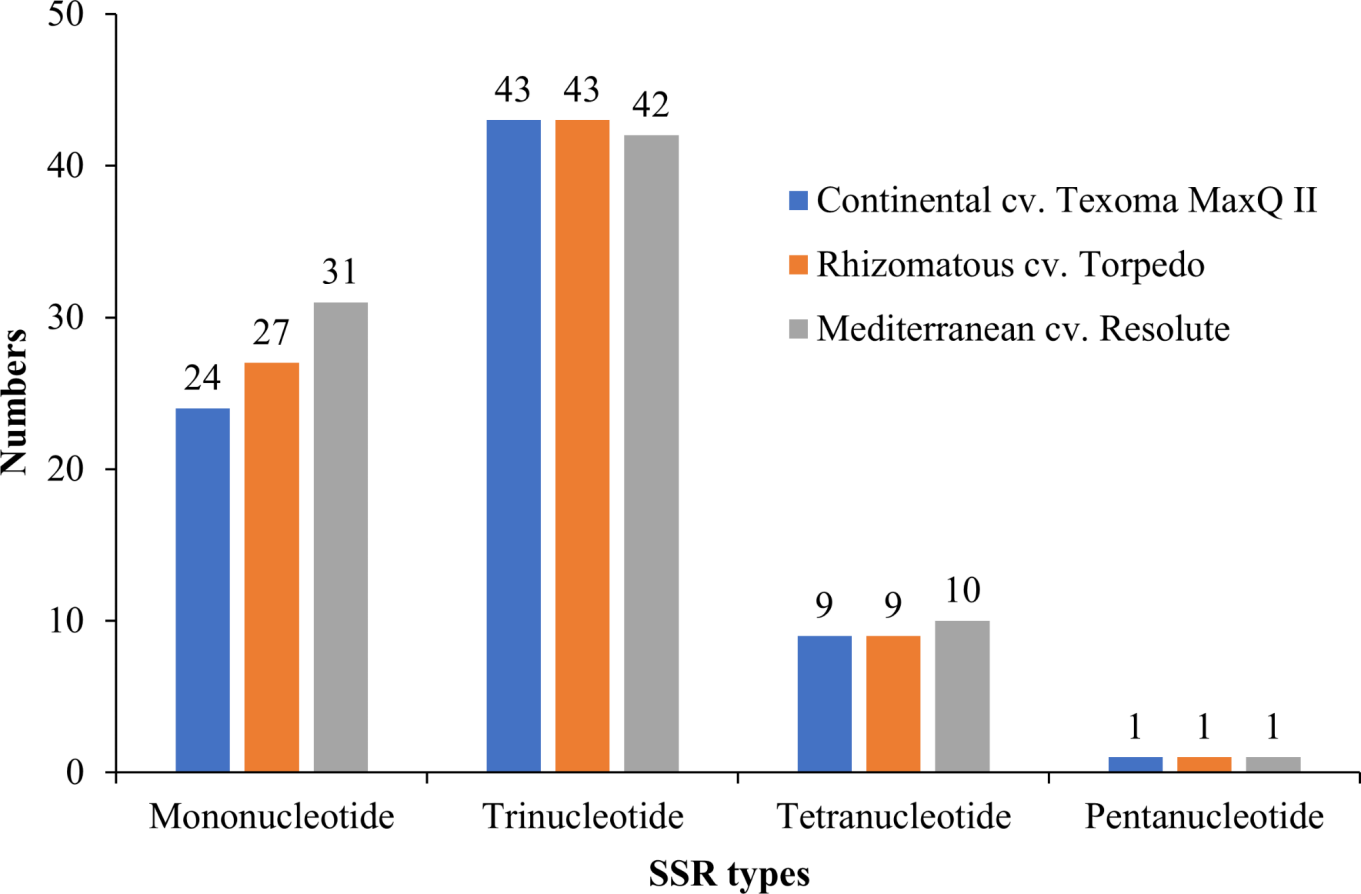
**Statistics of simple sequence repeats (SSRs) in the plastid genomes of tall fescue morphotypes**. The X-axis indicates the SSR types, and the Y-axis indicates the number of each SSR type in each morphotype plastid genome

We identified 15, 17, and 13 Tandem repeats (TRs) in Continental, Rhizomatous, and Mediterranean plastid genome, respectively with period size varied from 18 to 75 bp (Additional file 1: Table S4, Table S5, and Table S6). The average copy number of these TRs were 2.62, 2.54, and 2.52 in Continental, Rhizomatous, and Mediterranean morphotype’s plastid genome, respectively. A set of 77 SSRs (mono-, tri-, tetra-, and pentanucleotide repeats) specific to Continental, 80 SSRs specific to Rhizomatous, and 84 SSRs specific to Mediterranean plastid genomes with repeat lengths of at least nine nucleotides were identified. Among the SSR types, the highest (43) number of trinucleotide repeats were found in Continental and Rhizomatous and a single pentanucleotide repeat was found in all the three plastid genomes (Fig. 5, Additional file 1: Table S7, Table S8, and Table S9). The pentanucleotide repeat was 15 bp long in all plastid genomes. SSRs with dinucleotide repeats were not identified in any of the plastid genomes.

### Discovery of SNPs and InDels in plastid genomes using pairwise analysis

By making pairwise alignment, we identified 630 SNPs and 124 InDels between Continental and Mediterranean (Table 3 and Additional file 1: Table S10), 62 SNPs and 20 InDels between Continental and Rhizomatous (Table 3 and Additional file 1: Table S11), and 635 SNPs and 123 InDels between Rhizomatous and Mediterranean morphotype’s plastid genomes (Table 3 and Additional file 1: Table S12).

**Table 3:** Alignment length and number of SNPs and InDels identified in pairwise tall fescue morphotype’s plastid genomes

Tall fescue morphotypes^*^	Alignment lengthǂ (bp)	Number of SNPs	Number of InDels
Continental/Mediterranean	135,622	630	124
Continental/Rhizomatous	135,384	62	20
Rhizomatous/Mediterranean	135,683	635	123

^*^Continental cv. Texoma MaxQ II, Rhizomatous cv. Torpedo, and Mediterranean cv. Resolute

ǂThe alignment length between two genomes was increased from the actual genome size due to the presence of InDels in the genomes

**Fig. 6:**
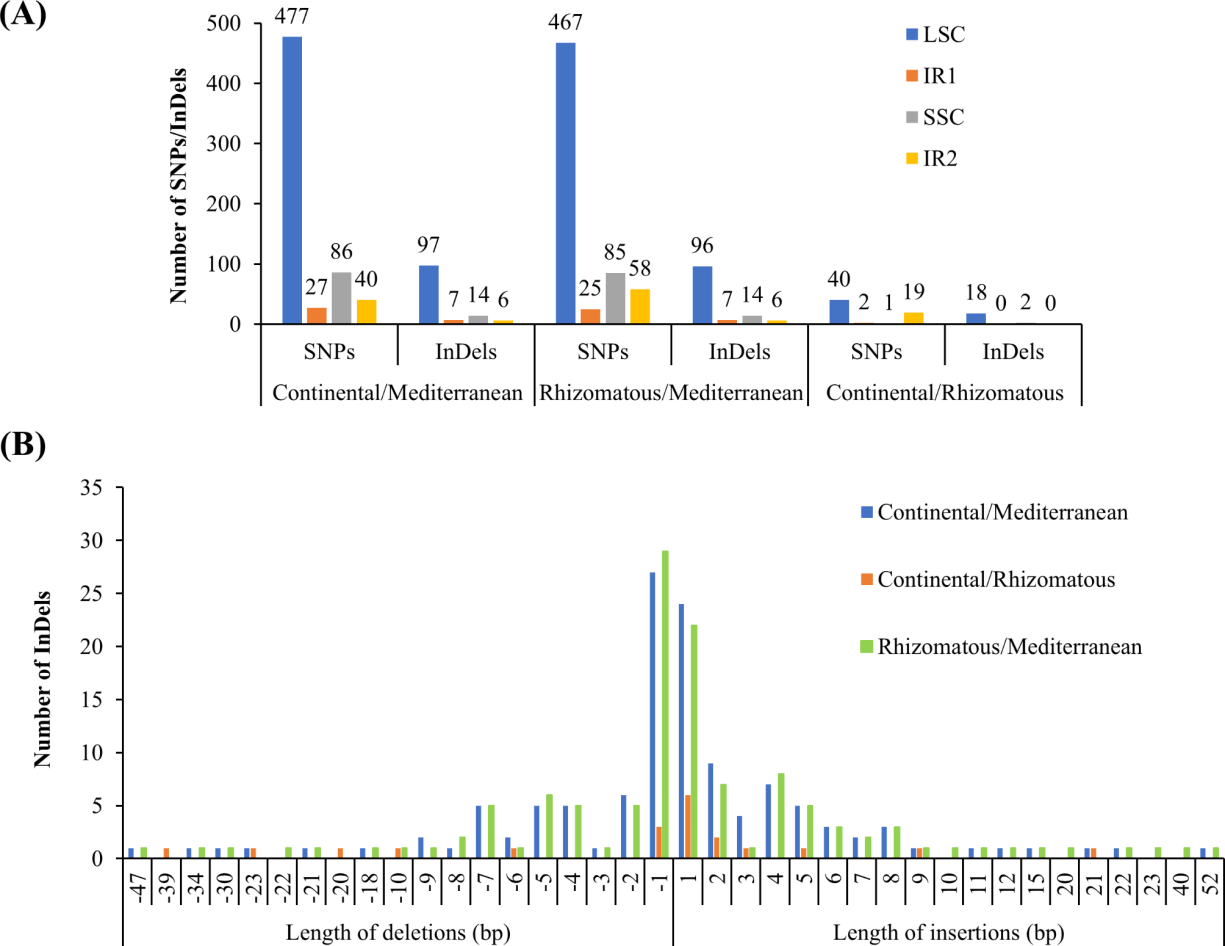
**Number and distribution of SNPs and InDels as well as length of InDels in pairwise comparisons**. (**A**) distribution of SNPs and InDels in the LSC, IRs, and SSC region, and (**B**) length of insertions and length of deletions identified in pairwise comparisons

The highest and lowest number of SNPs were found in LSC and IR1 regions, respectively in Continental/Mediterranean and Rhizomatous/Mediterranean. But the highest and lowest number of InDels were found in LSC and IR2 regions, respectively in Continental/Mediterranean and Rhizomatous/Mediterranean. In Continental/Rhizomatous, the highest (40) and lowest (one) number of SNPs were found in LSC and SSC regions, respectively. The highest number of InDels were found in LSC region, but no InDels were detected in both IR1 and IR2 regions in Continental/Rhizomatous (Fig. 6A).

The maximum number of SNPs (17) were identified at position 56,001–57,000 bp and 63,001–64,000 bp located in LSC region between Continental and Mediterranean plastid genomes, while the maximum number of SNPs (30) were identified between Rhizomatous and Mediterranean plastid genomes at position 122,001-123,000 bp located in IR2 region. The position 122,001-123,000 bp of IR2 region also contained maximum SNPs (19) between Continental and Rhizomatous plastid genomes (Additional file 2: Figure S1, Additional file 1: Table S10, Table S11, and Table S12).

In this study, we identified the highest length of insertion (52-bp) located in the SSC region and the highest length of deletion (47-bp) located in the IR1 region in Continental and Rhizomatous when compared to the Mediterranean plastid genome (Fig. 6B, Additional file 1: Table S10 and Table S12). Similarly, we identified a 20-bp and a 39-bp of deletions in the LSC region those made Continental sample unique from the Rhizomatous sample (Fig. 6B, Additional file 1: Table S11). The majority of the InDels were of single nucleotide in all three pairs (Fig. 6B).

### Variation of gene length in tall fescue morphotype’s plastid genomes

By examining the genic region of tall fescue morphotype’s plastid genome, we found gene length variation of 6-bp, 21-bp and 12-bp in three protein-coding genes (*Cytochrome c synthesis A*, *ccsA*; *ribosomal protein subunit 18*, *rps18*; and *Acetyl-coenzyme A carboxylase, carboxyl transferase subunit beta*, *accD*, respectively) and 9-bp in one pseudo protein-coding gene (*NADH dehydrogenase subunit H*, *ndhH-p*). In case of *accD* gene, we observed a SNP mutation (T→C) in the stop codon leading to the insertions of 12 bp results adding of four amino acids such as arginine, leucine, glutamic acid, and arginine in Mediterranean tall fescue in the 3ʹ region. These four InDels differentiate Mediterranean from Continental and Rhizomatous morphotypes (Table 4 and Additional file 2: Figure S2). The length of the remaining protein- and RNA-coding genes were found to be the same in all morphotypes (Additional file 1: Table S1, Table S2, and Table S3).

**Table 4:** Differences in the length of specific genes in the plastid genomes of tall fescue morphotypes

Name of the genes^a^	Length (bp)		
	Continental cv. Texoma MaxQ II	Rhizomatous cv. Torpedo	Mediterranean cv. Resolute
Cytochrome c synthesis A (*ccsA*)	960	960	966
Ribosomal protein subunit 18 (*rps18*)	450	450	471
Acetyl-coenzyme A carboxylase, carboxyl transferase subunit beta (*accD*)	153	153	165
NADH dehydrogenase subunit H *(ndhH*-p)	180	180	189

^a^p, pseudogene

### Development of tall fescue morphotype-specific InDel markers

**Fig. 7:**
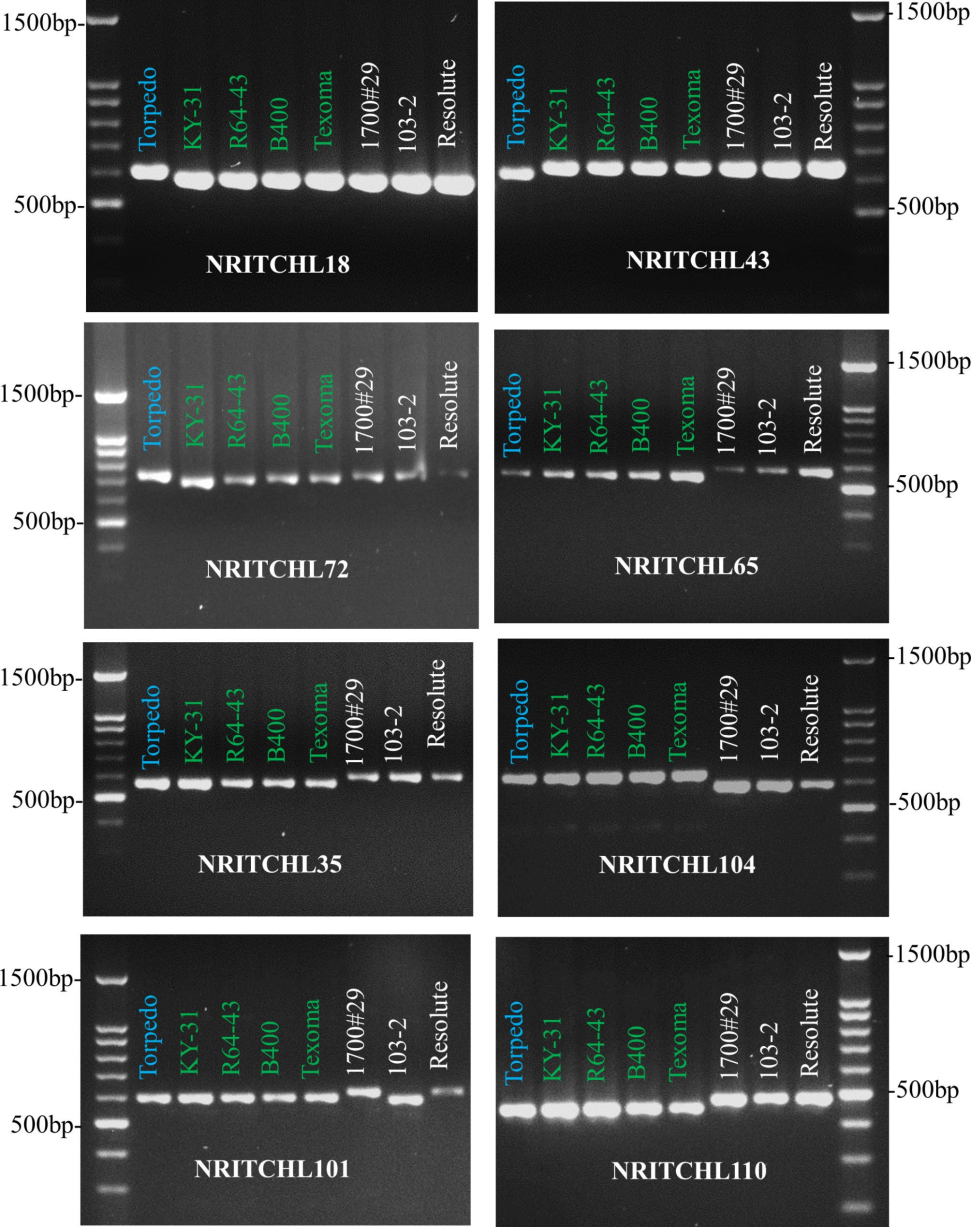
**PCR amplification of eight morphotype-specific DNA markers in eight tall fescue genotypes**. Torpedo belongs to Rhizomatous-, KY31, R64-43, B400, and Texoma MaxQ II belong to Continental- and 1700#29, 103-2, and Resolute belong to Mediterranean morphotype. The original and uncropped gel images were included in additional file 2: Figure S3

We developed plastid-derive eight InDel markers, namely, NRITCHL18, NRITCHL35, NRITCHL43, NRITCHL65, NRITCHL72, NRITCHL101, NRITCHL104, and NRITCHL110 as morphotype-specific DNA markers that were able to discriminate tall fescue morphotypes (Fig. 7; Table 5, and Additional file 2: Figure S3). Among them, three InDel markers, NRITCHL18, NRITCHL43, and NRITCHL72, discriminated Rhizomatous (Torpedo) from Continental (KY31, R43-64, B400 and Texoma) and Mediterranean (1700#29, 103-2 and Resolute) tall fescue. Similarly, four InDel markers, NRITCHL35, NRITCHL65, NRITCHL104, and NRITCHL110, discriminated Mediterranean from Continental and Rhizomatous tall fescue. More interestingly, one InDel marker, NRITCHL101, discriminated Mediterranean from Continental and Rhizomatous tall fescue as well as within genotypes of Mediterranean morphotype (Fig. 7). The marker, NRITCHL101 can also be used as genotype- and/or cultivar-specific marker within Mediterranean morphotype.

**Table 5:** Primers that are developed to discriminate the three tall fescue morphotypes

Marker name^*^	Forward primer (5’-3’)	Reverse primer (5’-3’)	Size (bp)		
			Resolute	Texoma	Torpedo
NRITCHL18	TGTGGAGAGGTAGATAACTAGATTGG	TTCCATTCCGGAGCATCTTA	582	587	610
NRITCHL35	CCCAGTGGAAGAACAGATAGC	CCACTAGCGGCTCCATACTC	598	564	564
NRITCHL43	TCCTTTTCCTTCTGCTTGGA	TACATCCCACAGTAATTTGC	646	645	624
NRITCHL65	CAAAGAAAGACTACTTCTTCTGGA	GGCCCTTTCGAATAAAACATC	583	553	554
NRITCHL72	CGGGTCCTTTATAGGGAAGG	TCATGATCAATTGCTTTCTGG	693	687	726
NRITCHL101	TCAATATGGGCCCTCAACAC	TCTTATATGGATCCCCTTATTCTTTTT	624	602	602
NRITCHL104	TTCCCTTCTTTTTATCCACTCG	GCGGATATAGGAGGGGTTTG	592	644	644
NRITCHL110	CGCTCCGGTGTAATTGATTT	CGCAGTTGGAATTTTGGAA	485	455	454

*Marker name: NRI, Noble Research Institute; T, tall fescue; CHL, chloroplast; and the last 2–3 digits number indicate the forward primer position (Kb) in the plastid genome

### Estimation of divergence time

**Fig. 8:**
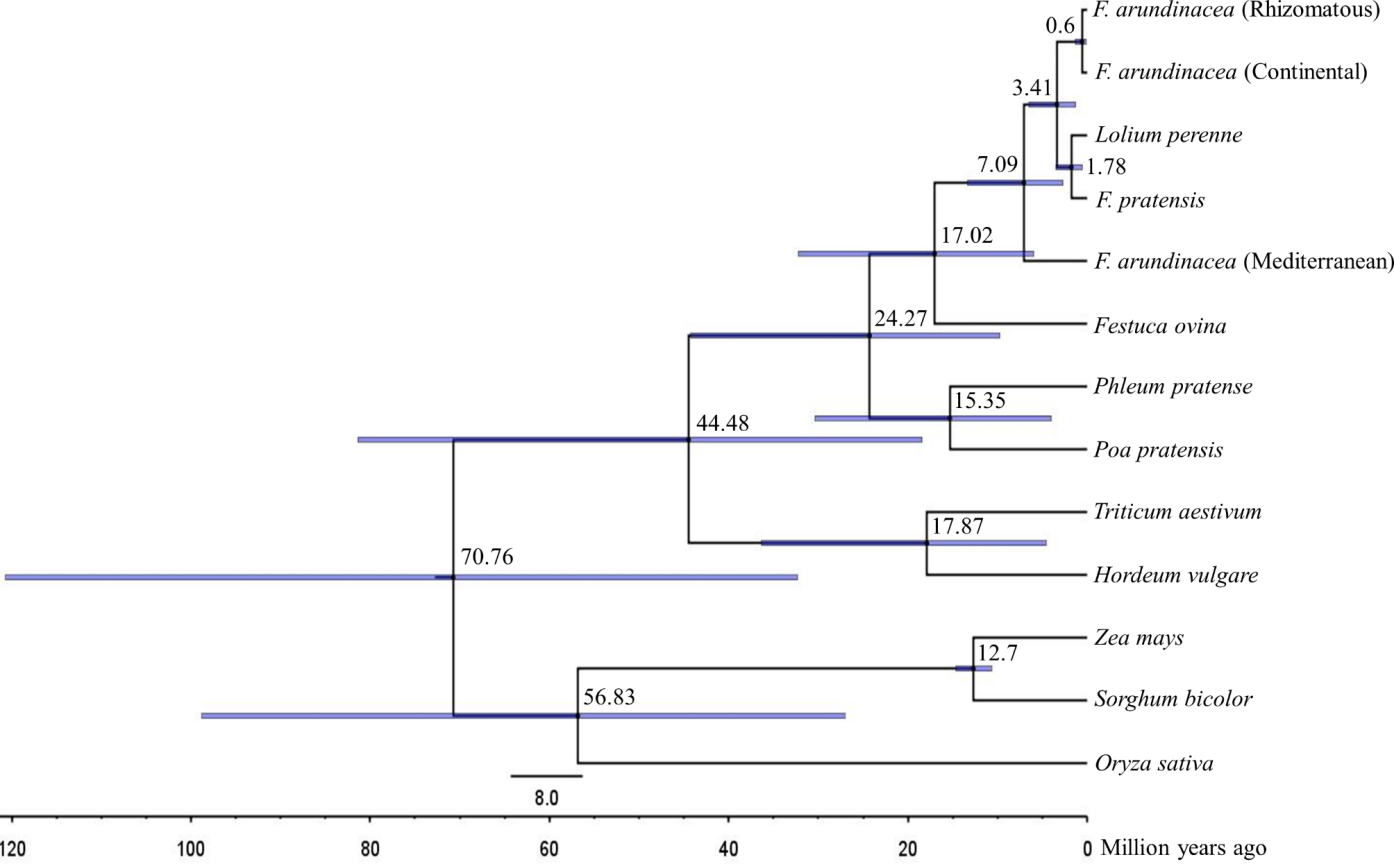
**Divergence time of tall fescue morphotypes along with 10 members of the Poaceae grass family based on the complete plastid genomes**. The numbers shown at each node indicate the divergence time and the blue bar represents a confidence interval of 95% highest posterior density

We used complete plastid genome sequences to analyze the divergence time between tall fescue morphotypes and closely related species of the Poaceae family (Additional file 1: Table S13). For the BEAST analysis, 13 complete plastid genomes were aligned for a total length of 150,130 bp. The Mediterranean tall fescue branched off from the Continental-, Rhizomatous tall fescue, perennial ryegrass (*Lolium perenne*), and meadow fescue (*F. pratensis*) approximately 7.09 million years ago (Mya) (95% HPD 2.69–13.38 Mya). The split time between Continental and Rhizomatous tall fescue was dated to about 0.6 (Mya) (95% HPD 0.15–1.35 Mya) (Fig. 8). Our results also showed that the divergence of *Festuca* was begun at approximately 17.02 Mya (95% HPD 5.99–32.23 Mya).

## Discussion

All three tall fescue morphotypes have distinct niches: Continental morphotype is suitable for forage production mainly in the Southeast and Midwest regions whereas Mediterranean has advantages in Southwestern region of USA, and Rhizomatous tall fescue is mainly used as turf grass. As genetic variation exists in tall fescue morphotypes, the complete sterility or low fertility often observed in hybrid progenies obtaining from crossing Continental with either Mediterranean or Rhizomatous tall fescue morphotypes [16–18]. This can be a consequence of crossing incompatibilities between tall fescue morphotypes [18]. To overcome the crossing incompatibility in breeding programs, it is necessary to identify tall fescue morphotypes using DNA markers during pre-breeding activities. The goal of this study was thus to sequence the plastid genomes of tall fescue morphotypes and to ascertain genomic variations and to identify morphotype-specific DNA markers, which will help to develop climate resilient smart cultivar(s). In this study, we reported the complete plastid genomes of three morphotypes of hexaploid tall fescue and compared the plastid genomes of the morphotypes. To our knowledge, this is the first report of the assembled plastid genomes of any Rhizomatous and Mediterranean tall fescue morphotypes. Our results are fundamental for future studies of gene editing into the plastid genome targeting photosynthetic mechanisms to increase forage and turf yield. Recently, Kang et al. [19] developed a Golden Gate cloning system for organelle DNA editing in plants, and generated lettuce antibiotic-resistant calli and plantlets with 99% edit frequencies by introducing a point mutation in the chloroplast 16 S rRNA gene.

Plastid genomes have been used to evaluate the genetic relatedness among the closely related plant species [20]. The tall fescue plastid genomes size ranges from 135,283 to 135,336 bp, suggesting the genetic differences among them are mainly due to SNPs and InDels variation in coding and non-coding regions. Though the KY31 and Texoma MaxQ II tall fescue belong to the same Continental morphotype, we observed that the Texoma MaxQ II plastid genome size was 765 bp smaller than that of KY31 (136,048 bp) [12]. KY31 originated in Menifee County, Kentucky, and is currently a commercial variety of tall fescue released in 1943 in the United States [21]. The differences in plastid genome size within Continental morphotype indicate that the KY31 might be genetically different or geographically isolated from other Continental tall fescue. The Continental tall fescue plastid genome size is close to the plastid genome size of diploid meadow fescue (135,291 bp) [22] and diploid perennial ryegrass (135,282 bp) [23].

Our comparative analysis among the three tall fescue plastid genomes showed highly conserved structures. In the quadripartite structure of plastid genomes, LSC, SSC, and two copies of identical IR were reported. The length of IR varied from 20,000 to 27,118 bp in higher plants [24]. The IR length variation might be due to the contraction and expansion events in the quadripartite structure, which induced length mutations in plastid genome in higher plants [25]. In our study, we observed that the position in the LSC/IR_1_, IR_1_/SSC, SSC/IR_2_ border regions was changed among the three tall fescue plastid genomes. The length of IRs in Continental and Rhizomatous was similar, but interestingly there was a 47-bp deletion in length between the two IRs in Mediterranean plastid genomes. This 47-bp deletion differentiates Continental- and Rhizomatous morphotypes from Mediterranean morphotype and is also identified by Talukder et al. [26] using NFTCHL45 marker. In our analysis, we found most of the SNPs and InDels in the LSC and SSC regions and few of them in the IR regions indicate that the IR regions are more conserved than the LSC and SSC regions.

Although the plastid genome is small in size in compared to its nuclear counter parts, repetitive sequences play a crucial role in the plastid genome arrangement and sequence divergence [27]. Due to a high rate of sequence rearrangement, the plastid derived SSRs as alternative to the nuclear SSR markers are useful for developing species-specific markers. The repeats in SSRs in tall fescue plastid genome were mostly comprised of adenine (A) or thymine (T) repeats but were rarely guanine (G) or cytosine (C) repeats (Additional file 1: Table S7, Table S8, and Table S9), which resulted lower GC content (38%) in tall fescue plastid genomes. These results are identical to other forage species [22, 23].

We identified an equal number of protein-, rRNA-, and tRNA-coding genes in the three morphotype’s plastid genomes. In the earlier study, the gene *ycf3* has been annotated as a hypothetical protein-coding gene in tall fescue [12], but we annotated *ycf3* gene as a protein-coding gene in all three morphotypes. Because the *ycf3* is essential for the assembly of the photosystem I (PSI) complex in the green plants [28–30]. Our annotation also discovered two regions (*ndhH-p* and *rpl23-p*) as pseudo protein-coding genes because they were highly homologous to the 5ʹ region and truncated to the 3ʹ region of the protein coding genes, *ndhH* and *rpl23*, respectively. The pseudo protein-coding genes, *ndhH-p* and *rpl23-p* probably unable to produce functional protein due to presence of immature stop codon at the 3ʹ region. Further studies are needed to understand the necessity of the pseudo genes in the plastid genome. In the tall fescue plastid genomes, we also found three highly variable regions (*ycf1*, *ycf2*, and y*cf68*), and annotated them until the first stop codon as hypothetical protein-coding genes. In earlier studies, they were not annotated as gene because they contained several internal stop codons in the coding regions and unknown functionality in different species [24, 31–33].

The plastid acetyl-CoA carboxylase (ACCase) complex is composed of four subunits, each encoded by a separate gene, such as biotin carboxylase (*accC*), biotin carboxyl carrier protein (*accB* = *bccp*), alpha-carboxyl transferase (*accA*), and beta-carboxyl transferase (*accD*). Out of the four genes, only *accD* gene is encoded in plastid genome in tall fescue morphotypes, while the remaining three genes are encoded in the nuclear genome.

The repetitive regions in the plastid genome are essential to induce SNPs and InDels. The majority of the InDels were located in the intergenic regions resulting in the lengthening and shortening of the plastid genome size. We found the low number of SNPs (62) and InDels (20) between Continental and Rhizomatous morphotypes suggests that they are probably evolved from the closely related parents, which in agreement with the findings of Hand et al. [1]. On the other hand, we obtained a higher number of SNPs and InDels between Continental and Mediterranean (630 SNPs and 124 InDels) and between Rhizomatous and Mediterranean (635 SNPs and 123 InDels) morphotypes, possibly indicating that the Mediterranean tall fescue is evolved from distantly related parents. In a previous study, Talukder et al. [26] showed that Mediterranean cultivars (Resolute, Flecha Max Q, and Prosper) clustered into a same group with Tetraploid tall fescue (*F. mairei*) accessions (PI 283312 and PI 283313, whose genetic constitution is M_1_M_1_M_2_M_2_) using chloroplast DNA sequence, while they clustered differently using nuclear genome derived SSR marker analysis. By analyzing chloroplast DNA sequence, EST- and genomic-based SSRs, and ITS-derived data, Talukder et al. [26] and Hand et al. [1] reported the independent evolution of Continental and Mediterranean morphotypes. The divergence time tree in our study showed that the Mediterranean tall fescue evolved 7.09 Mya, while the Continental- and Rhizomatous tall fescue evolved only 0.6 Mya (Fig. 8). The SNPs and InDels data as well as the divergence time suggests Mediterranean tall fescue evolved much earlier than Continental- and Rhizomatous tall fescue. However, the continental- and Rhizomatous tall fescue evolved after a recent diversification of *Festuca* genus. Divergence of other members within the Poaceae family analyzed in this study followed the same estimation as reported in previous studies [34–37].

## Conclusions

The complete plastid genome sequences constitute the important genomic resources for future studies in the field of genetic engineering in photosynthetic mechanism of tall fescue morphotypes. By comparing the whole plastid genome, we identified SNPs and InDels using pairwise alignment. From the InDel variants, we developed eight InDels markers to discriminate tall fescue morphotypes. Divergence time estimation revealed that Mediterranean tall fescue evolved much earlier than the other two morphotypes. The data presented in this study would be helpful to improve our understanding of the evolutionary genetics and biology of the *Festuca* species.

## Methods

### Plant material and isolation of chloroplast DNA

One cultivar of each tall fescue morphotype, such as Continental cv. Texoma MaxQ II, Rhizomatous cv. Torpedo, and Mediterranean cv. Resolute were grown in gallon pots (15.87 cm x 16.51 cm) at the Noble Research Institute’s greenhouse, Ardmore, Oklahoma. The Continental tall fescue cv. Texoma MaxQ II was developed for improved persistence and forage yield at the Noble Research Institute, Oklahoma, United States [38] and is freely available for cultivation in the south-central United States (www.pennington.com). The Mediterranean cv. Resolute was developed through phenotypic selection from an open pollinated field of cv. Melik for improved forage and seed production [39]. The Rhizomatous cv. Torpedo was selected from winter active rhizomatous germplasms collected from Galicia, Spain [40]. Seeds of Rhizomatous and Mediterranean morphotypes were collected from PGG Wrightson Seeds, New Zealand (https://pggwrightsonseeds.com/). Chloroplast DNA was extracted from 40 g fresh green leaf tissues collected from eight-week-old plants according to the protocol developed by Islam et al. [14].

### Sequencing and reference assembly of tall fescue plastid genomes

Tall fescue plastid genomes of Continental-, Rhizomatous-, and Mediterranean morphotypes were sequenced using an Illumina MiSeq paired-end (2 × 300 bp) sequencing technology. The filtering procedures of raw sequence reads and contaminating mitochondrial reads have been described [14]. The filtered reads were assembled in reference-guided assemblies against the plastid genome sequences of KY31 (GenBank Acc. No.: FJ466687) [12] using Qiagen CLC Genomics Workbench (v.12.0) (https://digitalinsights.qiagen.com/) using the default parameters. The reference assembly produced several gaps between the reference and consensus sequences. PCR primers spanning the gap regions were designed using the web interface of Primer3 Input software (v.0.4.0) (https://bioinfo.ut.ee/primer3-0.4.0/) to close the gaps via PCR amplification. Genomic DNA was used as template for PCR amplification. The purified PCR products were sequenced at the Genomics Core Facility in the Noble Research Institute. The gap sequences were then included into the final assembly using the SeqMan Pro (v.16.0) software (DNASTAR, Madison, WI).

### Plastid genome annotation

The assembled plastid genome of each morphotype was annotated using web interface of GeSeq software pipeline [41] with the following parameters: sequence source, land plant’s plastid; annotate plastid IR, yes; annotate plastid trans-spliced *rps12*, yes; protein search identity, 25; rRNA, tRNA, and DNA search identity, 85; added NCBI RefSeq(s)- *F. altissima*, *F*. *ovina*, *F*. *pratensis*, *Lolium arundinaceum*, *L*. *perenne*, *L*. *multiflorum*; MPI-MP reference set, chloroplast land plants (CDS + rRNA), 3rd party stand-alone annotators, Chloë v.0.1.0 (CDS, tRNA and rRNA). The resulting annotated file (GFF3) containing all genes encoding proteins, rRNAs, and tRNAs was loaded into Geneious Prime software (v.2019.0.4) (https://www.geneious.com) for visualization and manual inspection of the genes. The plastid genome map of each morphotype was drawn using the web-based software Proksee (https://proksee.ca/) (Date of access: October 27, 2022) [42].

### Structural variation analysis

Tandem repeats (TRs) were investigated using the Tandem Repeat Finder (v.4.09) with advance parameter settings (alignment parameters [match, 2; mismatch, 7; InDel, 7]; minimum alignment score to report repeat, 80; maximum period size, 2,000; maximum TR array size, 1 million bp) [43]. Microsatellites were detected using the web interface of MISA software [44]. The parameters for microsatellite detection were 1- to 2-nucleotide repeats of at least 10 nucleotide length and 3- to 6-nucleotide repeats with at least three repeat units. Large IRs of each plastid genome were identified by nucleotide BLAST program (https://blast.ncbi.nlm.nih.gov/Blast.cgi).

### Analysis of SNPs and InDels

The plastid genome sequences of Continental-, Rhizomatous- and Mediterranean morphotypes were aligned pairwise using the web server of Clustal Omega sequence alignment program (https://www.ebi.ac.uk/Tools/msa/clustalo/) with default parameters settings. The aligned sequences were saved as NEXUS file format. The polymorphic sites such as SNPs and InDels (gap options: Model 1- Diallelic) between morphotype plastid genomes were analyzed using DnaSP (v. 6) software [45].

### Development of tall fescue morphotype-specific InDel markers

After performing the alignment of plastid genome sequences of the three tall fescue morphotypes using CLC Genomics Workbench (v.12.0) software (https://digitalinsights.qiagen.com/) with default parameter settings, InDel regions were identified. To confirm the identified InDel regions, PCR primers were designed from the flanking sequences spanning the InDel regions using the Primer3 Input software (v.0.4.0) (https://bioinfo.ut.ee/primer3-0.4.0/). PCR amplifications and DNA band visualization were performed using the above-mentioned protocols.

### Estimation of divergence time

A divergence time tree was constructed using 13 plastid genomes within the Poaceae grass family (3 from this study and 10 previously published) which includes an outgroup member from Oryzoideae and two from Panicoideae subfamilies (Additional file 1: Table S13). The 13 complete chloroplast genomes were then aligned using the default parameter setting of the MAFFT program implemented in the DNASTAR Lasergene (v.17.4.2) (DNASTAR, Madison, WI). We then estimated the divergence time of tall fescue morphotypes along with 10 members of the Poaceae family using the Bayesian relaxed clock model in BEAST (v.1.10.4) [46]. The GTR + G nucleotide substitution model, estimated base frequencies, the uncorrelated relaxed lognormal clock model, and the birth-death process were used as the tree prior to the BEAST analysis. The monophyletic constraint was enforced during specifying a taxon set to calibrate the evolutionary rates. Normal prior distribution was used to calibrate the split time (13.0 ± 1.0 Mya) between *Zea mays* and *Sorghum bicolor* [35]. Two independent Markov chain Monte Carlo (MCMC) runs were performed with each chain running for 100,000,000 generations and sampling every 10,000 generations. An effective sample size (ESS) > 200 for all relevant parameters and convergence of the MCMC chains were evaluated by Tracer (v.1.7.2) [47]. LogCombiner v.1.10.4 (https://beast.community/logcombiner) was used to combine the tree files generated from the two independent MCMC runs and the first 20% of trees were removed as burn-in from the combined tree file. The maximum clade credibility tree was then constructed with median node height and posterior probability limit of 0.5 using TreeAnnotator v.1.10.4 (https://beast.community/treeannotator). FigTree v.1.4.4 (http://tree.bio.ed.ac.uk/software/figtree/) was used to visualize the divergence time of each node with the confidence interval of highest posterior density (HPD) at 95%.

### Electronic supplementary material

Below is the link to the electronic supplementary material.

Additional file 1: Tables S1-S13

Additional file 2: Figs. S1-S3

## Data Availability

The complete assembled plastid genome sequences of the Continental, Mediterranean, and Rhizomatous tall fescue morphotypes have been deposited to the National Center for Biotechnology Information (NCBI) GenBank under the accession number OQ928086, OQ935426, and OQ935427, respectively.

## List of abbreviations

BLAST: Basic local alignment search tool
bp: Base pair
CDS: Coding sequence
CV: Cultivar
DNA: Deoxyribonucleic acid
EST: Expressed sequence tag
g: Gram
InDels: Insertions and deletions
IR: Inverted repeat
ITS: Internal transcribed region
Kb: Kilo base pair
LSC: Large single copy
Mya: Million years ago
PCR: Polymerase chain reaction
RNA: Ribonucleic acid
rRNA: Ribosomal RNA
SNPs: Single nucleotide polymorphisms
SSC: Small single copy
TR: Tandem repeat
tRNA: Transfer RNA
U: Unit
