# Supplementary material for: Dig up tall fescue plastid genomes for the identification of morphotype-specific DNA variants
Source: BMC Genomics. 2023 Oct 3;24:586. doi: 10.1186/s12864-023-09631-8 (PMC10546690; doi:10.1186/s12864-023-09631-8)
Supplement: Supplementary file 1 — Additional file 1: Tables S1-S13 [file 12864_2023_9631_MOESM1_ESM.zip › Additional file 1 Table S1_updated_ESM.docx]

**Additional file 1: Table S1**. Genes identified in Continental cv. Texoma MaxQ II tall fescue plastid genome.

A.

| Protein-coding genes^a^ | Length^b^ (bp) | Position in the genome (bp) | | Direction^c^ | Number of amino acids |
| --- | --- | --- | --- | --- | --- |
|  |  | From | To |  |  |
| 1. Subunits of photosystem I | | | | | |
| *psaA* | 2,253 | 38,697 | 40,949 | - | 750 |
| *psaB* | 2,205 | 36,467 | 38,671 | - | 734 |
| *psaC* | 246 | 108,200 | 108,445 | - | 81 |
| *psaI* | 111 | 56,911 | 57,021 | + | 36 |
| *psaJ* | 129 | 64,202 | 64,330 | + | 42 |
| 2. Assembly factors of photosystem I | | | | | |
| *ycf3* | **519** |  |  | - | **172** |
| *ycf3a* | 132 | 43,462 | 43,593 | - | 44 |
| *ycf3b* | 226 | 42,488 | 42,713 | - | 75 |
| *ycf3c* | 161 | 41,604 | 41,764 | - | 53 |
| *ycf4* | 558 | 57,330 | 57,887 | + | 185 |
| 3. Subunits of photosystem II | | | | | |
| *psbA* | 1,062 | 88 | 1,149 | - | 353 |
| *psbB* | 1,527 | 68,402 | 69,928 | + | 508 |
| *psbC* | 1,422 | 9,740 | 11,161 | + | 473 |
| *psbD* | 1,062 | 8,731 | 9,792 | + | 353 |
| *psbE* | 252 | 61,539 | 61,790 | - | 83 |
| *psbF* | 120 | 61,409 | 61,528 | - | 39 |
| *psbH* | 222 | 70,497 | 70,718 | + | 73 |
| *psbI* | 111 | 7,428 | 7,538 | + | 36 |
| *psbJ* | 123 | 61,021 | 61,143 | - | 40 |
| *psbK* | 186 | 6,840 | 7,025 | + | 61 |
| *psbL* | 117 | 61,270 | 61,386 | - | 38 |
| *psbM* | 105 | 16,597 | 16,701 | + | 34 |
| *psbT* | 117 | 70,097 | 70,213 | ­+ | 38 |
| *psbZ* | 189 | 11,759 | 11,947 | + | 62 |
| 4. Subunits of the cytochrome b_6_/f complex | | | | | |
| *petA* | 963 | 59,262 | 60,224 | + | 320 |
| *petB* | **648** |  |  | + | **215** |
| *petBa* | 6 | 70,848 | 70,853 | + | 2 |
| *petBb* | 642 | 71,606 | 72,247 | + | 213 |
| *petD* | **483** |  |  |  | **160** |
| *petDa* | 8 | 72,438 | 72,445 | + | 2 |
| *petDb* | 475 | 73,123 | 73,597 | + | 158 |
| *petG* | 114 | 63,341 | 63,454 | + | 37 |
| *petL* | 96 | 63,072 | 63,167 | + | 31 |
| *petN* | 90 | 16,980 | 17,069 | - | 29 |
| 5. Cytochrome c synthesis | | | | | |
| *ccsA* | 960 | 105,470 | 106,429 | + | 319 |
| 6. Photosystem biogenesis factor 1 | | | | | |
| *pbf1* | 132 | 70,262 | 70,393 | - | 43 |
| 7. Subunits of ATP synthase | | | | | |
| *atpA* | 1,524 | 33,923 | 35,446 | + | 507 |
| *atpB* | 1,497 | 51,999 | 53,495 | - | 498 |
| *atpE* | 414 | 51,589 | 52,002 | - | 137 |
| *atpF* | **567** |  |  | + | **188** |
| *atpFa* | 158 | 32,448 | 32,605 | + | 52 |
| *atpFb* | 409 | 33,423 | 33,831 | + | 136 |
| *atpH* | 246 | 31,740 | 31,985 | + | 81 |
| *atpI* | 744 | 30,432 | 31,175 | + | 247 |
| 8. Subunits of NADH-dehydrogenase | | | | | |
| *ndhA* | **1,089** |  |  | - | **362** |
| *ndhAa* | 550 | 112,306 | 112,855 | - | 183 |
| *ndhAb* | 539 | 110,746 | 111,284 | - | 179 |
| *ndhB-1* | **1,533** |  |  | - | **510** |
| *ndhB-1a* | 777 | 87,100 | 87,876 | - | 259 |
| *ndhB-1b* | 756 | 85,629 | 86,384 | - | 251 |
| *ndhB-2* | **1,533** |  |  | + | **510** |
| *ndhB-2a* | 777 | 127,361 | 128,137 | + | 259 |
| *ndhB-2b* | 756 | 128,853 | 129,608 | + | 251 |
| *ndhC* | 363 | 49,335 | 49,697 | - | 120 |
| *ndhD* | 1,509 | 106,572 | 108,080 | - | 502 |
| *ndhE* | 306 | 108,944 | 109,249 | - | 101 |
| *ndhF* | 2,226 | 101,473 | 103,698 | - | 741 |
| *ndhG* | 531 | 109,462 | 109,992 | - | 176 |
| *ndhH* | 1,182 | 112,857 | 114,038 | - | 393 |
| *ndhI* | 543 | 110,108 | 110,650 | - | 180 |
| *ndhJ* | 480 | 48,025 | 48,504 | - | 159 |
| *ndhK* | 741 | 48,604 | 49,344 | - | 246 |
| 9. Large subunit of RubisCo | | | | | |
| *rbcL* | 1,434 | 54,290 | 55,723 | + | 477 |
| 10. Subunits of the DNA-dependent RNA polymerase | | | | | |
| *rpoA* | 1,026 | 73,808 | 74,833 | - | 341 |
| *rpoB* | 3,231 | 19,253 | 22,483 | + | 1076 |
| *rpoC1* | 2,031 | 22,521 | 24,551 | + | 676 |
| *rpoC2* | 4,422 | 24,755 | 29,176 | + | 1473 |
| 11. Small subunits of ribosomal proteins | | | | | |
| *rps2* | 711 | 29,467 | 30,177 | + | 236 |
| *rps3* | 720 | 78,675 | 79,394 | - | 239 |
| *rps4* | 606 | 44,563 | 45,168 | - | 201 |
| *rps7-1* | 471 | 88,176 | 88,646 | - | 156 |
| *rps7-2* | 471 | 126,591 | 127,061 | + | 156 |
| *rps8* | 411 | 76,166 | 76,576 | - | 136 |
| *rps11* | 432 | 74,898 | 75,329 | - | 143 |
| *rps12-1* | **375** |  |  | x | **124** |
| *rps12-1a* | 114 | 66,930 | 67,043 | - | 38 |
| *rps12-1b* | 232 | 89,269 | 89,500 | - | 77 |
| *rps12-1c* | 29 | 88,700 | 88,728 | - | 9 |
| *rps12-2* | **375** |  |  | x | **124** |
| *rps12-2a* | 114 | 66,930 | 67,043 | - | 38 |
| *rps12-2b* | 232 | 125,737 | 125,968 | + | 77 |
| *rps12-2c* | 29 | 126,509 | 126,537 | + | 9 |
| *rps14* | 312 | 35,995 | 36,306 | - | 103 |
| *rps15-1* | 273 | 100,797 | 101,069 | + | 90 |
| *rps15-2* | 273 | 114,168 | 114,440 | - | 90 |
| *rps16* | **270** |  |  | - | **89** |
| *rps16a* | 40 | 5,603 | 5,642 | - | 13 |
| *rps16b* | 230 | 4,531 | 4,760 | - | 76 |
| *rps18* | 450 | 65,261 | 65,710 | + | 149 |
| *rps19-1* | 267 | 79,989 | 80,255 | - | 88 |
| *rps19-2* | 267 | 134,982 | 135,248 | + | 88 |
| 12. Large subunits of ribosomal proteins | | | | | |
| *rpl2-1* | **822** |  |  | - | **273** |
| *rpl2-1a* | 391 | 81,627 | 82,017 | - | 130 |
| *rpl2-1b* | 431 | 80,533 | 80,963 | - | 143 |
| *rpl2-2* | **822** |  |  | + | **273** |
| *rpl2-2a* | 391 | 133,220 | 133,610 | + | 130 |
| *rpl2-2b* | 431 | 134,274 | 134,704 | + | 143 |
| *rpl14* | 372 | 76,720 | 77,091 | - | 123 |
| *rpl16* | **411** |  |  | - | **136** |
| *rpl16a* | 9 | 78,503 | 78,511 | - | 3 |
| *rpl16b* | 402 | 77,213 | 77,614 | - | 133 |
| *rpl20* | 360 | 65,873 | 66,232 | - | 119 |
| *rpl22* | 444 | 79,467 | 79,910 | - | 147 |
| *rpl23-1* | 282 | 82,036 | 82,317 | - | 93 |
| *rpl23-2* | 282 | 132,920 | 133,201 | + | 93 |
| *rpl32* | 180 | 104,470 | 104,649 | + | 59 |
| *rpl33* | 201 | 64,762 | 64,962 | + | 66 |
| *rpl36* | 114 | 75,523 | 75,636 | - | 37 |
| 13. Translational initiation factor I | | | | | |
| *infA* | 342 | 75,742 | 76,083 | - | 113 |
| 14. Other protein coding genes | | | | | |
| Maturase (*matK*) | 1,536 | 1,697 | 3,232 | - | 511 |
| Envelope membrane protein (*cemA*) | 693 | 58,347 | 59,039 | + | 230 |
| Acetyl-coenzyme A carboxylase carboxyl transferase subunit beta (*accD*) | 153 | 56,474 | 56,626 | + | 50 |
| Clp protease proteolytic subunit (*clpP1*) | 651 | 67,185 | 67,835 | - | 216 |
| 15. Pseudo protein-coding genes | | | | | |
| *ndhH-p* | 180 | 101,199 | 101,378 | + | 59 |
| *rpl23-p* | 72 | 56,004 | 56,075 | + | 23 |
| 16. Hypothetical protein-coding genes | | | | | |
| *ycf1-1* | 120 | 99,594 | 99,713 | + | 39 |
| *ycf1-2* | 120 | 115,524 | 115,643 | - | 39 |
| *ycf2-1* | 96 | 82,658 | 82,753 | + | 31 |
| *ycf2-2* | 96 | 132,484 | 132,579 | - | 31 |
| *ycf68-1* | 381 | 93,373 | 93,753 | + | 126 |
| *ycf68-2* | 381 | 121,484 | 121,864 | - | 126 |

B.

| rRNA-coding genes^a^ | Length^b^ (bp) | Position in the genome (bp) | | Direction^c^ | Number of amino acids |
| --- | --- | --- | --- | --- | --- |
|  |  | From | To |  |  |
| *rrn4.5-1* | 95 | 98,188 | 98,282 | + |  |
| *rrn4.5-2* | 95 | 116,955 | 117,049 | - |  |
| *rrn5-1* | 121 | 98,510 | 98,630 | + |  |
| *rrn5-2* | 121 | 116,607 | 116,727 | - |  |
| *rrn16-1* | 1,492 | 91,427 | 92,918 | + |  |
| *rrn16-2* | 1,492 | 122,319 | 123,810 | - |  |
| *rrn23-1* | 2,889 | 95,205 | 98,093 | + |  |
| *rrn23-2* | 2,889 | 117,144 | 120,032 | - |  |


| tRNA-coding genes^a^ | Length^b^ (bp) | Position in the genome (bp) | | Direction^c^ | tRNA type | Anti-codon |
| --- | --- | --- | --- | --- | --- | --- |
|  |  | From | To |  |  |  |
| *trnA-1* | **73** |  |  | + | Ala | UGC |
| *trnA-1a* | 38 | 94,176 | 94,213 | + |  |  |
| *trnA-1b* | 35 | 95,025 | 95,059 | + |  |  |
| *trnA-2* | **73** |  |  | - | Ala | UGC |
| *trnA-2a* | 38 | 121,024 | 121,061 | - |  |  |
| *trnA-2b* | 35 | 120,178 | 120,212 | - |  |  |
| *trnC* | 71 | 17,994 | 18,064 | - | Cys | GCA |
| *trnD* | 74 | 16,050 | 16,123 | + | Asp | GUC |
| *trnE* | 73 | 15,469 | 15,541 | + | Glu | UUC |
| *trnF* | 73 | 47,368 | 47,440 | + | Phe | GAA |
| *trnG-1* | 71 | 12,229 | 12,299 | + | Gly | GCC |
| *trnG-2* | **71** |  |  | - | Gly | UCC |
| *trnG-2a* | 23 | 13,669 | 13,691 | - |  |  |
| *trnG-2b* | 48 | 12,943 | 12,990 | - |  |  |
| *trnH-1* | 75 | 80,403 | 80,477 | + | His | GUG |
| *trnH-2* | 75 | 134,760 | 134,834 | - | His | GUG |
| *trnI-1* | 74 | 82,492 | 82,565 | - | Ile | CAU |
| *trnI-2* | **77** |  |  | + | Ile | GAU |
| *trnI-2a* | 42 | 93,232 | 93,273 | + |  |  |
| *trnI-2b* | 35 | 94,076 | 94,110 | + |  |  |
| *trnI-3* | **77** |  |  | - | Ile | GAU |
| *trnI-3a* | 42 | 121,964 | 122,005 | - |  |  |
| *trnI-3b* | 35 | 121,127 | 121,161 | - |  |  |
| *trnI-4* | 74 | 132,672 | 132,745 | + | Ile | CAU |
| *trnK* | **72** |  |  | - | Lys | UUU |
| *trnK-a* | 37 | 3,916 | 3,952 | - |  |  |
| *trnK-b* | 35 | 1,384 | 1,418 | - |  |  |
| *trnL-1* | **85** |  |  | + | Leu | UAA |
| *trnL-1a* | 35 | 46,392 | 46,426 | + |  |  |
| *trnL-1b* | 50 | 46,977 | 47,026 | + |  |  |
| *trnL-2* | 81 | 85,016 | 85,096 | - | Leu | CAA |
| *trnL-3* | 80 | 105,313 | 105,392 | + | Leu | UAG |
| *trnL-4* | 81 | 130,141 | 130,221 | + | Leu | CAA |
| *trnfM* | 74 | 12,779 | 12,852 | - | Met | CAU |
| *trnM-1* | 59 | 14,865 | 14,923 | + | Met | CAU |
| *trnM-2* | 71 | 45489 | 45559 | - | Met | CAU |
| *trnM-3* | 73 | 51,402 | 51,474 | + | Met | CAU |
| *trnN-1* | 72 | 99,185 | 99,257 | - | Asn | GUU |
| *trnN-2* | 73 | 115,980 | 116,052 | + | Asn | GUU |
| *trnP* | 75 | 63,791 | 63,865 | - | Pro | UGG |
| *trnQ* | 73 | 6,424 | 6,496 | - | Gln | UUG |
| *trnR-1* | 72 | 35,544 | 35,615 | - | Arg | UCU |
| *trnR-2* | 74 | 98,859 | 98,932 | + | Arg | ACG |
| *trnR-3* | 74 | 116,305 | 116,378 | - | Arg | ACG |
| *trnS-1* | 88 | 7,657 | 7,744 | - | Ser | GCU |
| *trnS-2* | 88 | 11,317 | 11,404 | - | Ser | UGA |
| *trnS-3* | 87 | 44,211 | 44,297 | + | Ser | GGA |
| *trnT-1* | 72 | 14,859 | 14,930 | + | Thr | GGU |
| *trnT-2* | 61 | 14,931 | 14,991 | + | Thr | GGU |
| *trnT-3* | 73 | 45,487 | 45,559 | - | Thr | UGU |
| *trnV-1* | **76** |  |  | - | Val | UAC |
| *trnV-1a* | 39 | 51,177 | 51,215 | - |  |  |
| *trnV-1b* | 37 | 50,537 | 50,573 | - |  |  |
| *trnV-2* | 72 | 91,126 | 91,197 | + | Val | GAC |
| *trnV-3* | 72 | 124,040 | 124,111 | - | Val | GAC |
| *trnW* | 74 | 63,577 | 63,650 | - | Trp | CCA |
| *trnY* | 84 | 15,603 | 15,686 | + | Tyr | GUA |

^a^p, pseudogene; ^b^Boldface, sum of all exons; lower-case letters, exon of genes; hyphenated, duplicate genes; ^c^Plus and minus, forward and reverse DNA strand, respectively; x, trans-spliced.
