# Supplementary material for: Dig up tall fescue plastid genomes for the identification of morphotype-specific DNA variants
Source: BMC Genomics. 2023 Oct 3;24:586. doi: 10.1186/s12864-023-09631-8 (PMC10546690; doi:10.1186/s12864-023-09631-8)
Supplement: Supplementary file 1 — Additional file 1: Tables S1-S13 [file 12864_2023_9631_MOESM1_ESM.zip › Additional file 1 Table S2_updated_ESM.docx]

**Additional file 1: Table S2.** Genes identified in Rhizomatous cv. Torpedo tall fescue plastid genome.

A.

| Protein-coding genes^a^ | Length^b^ (bp) | Position in the genome (bp) | | Direction^c^ | Number of amino acids |
| --- | --- | --- | --- | --- | --- |
|  |  | From | To |  |  |
| 1. Subunits of photosystem I | | | | | |
| *psaA* | 2,253 | 38,706 | 40,958 | - | 750 |
| *psaB* | 2,205 | 36,476 | 38,680 | - | 734 |
| *psaC* | 246 | 108,254 | 108,499 | - | 81 |
| *psaI* | 111 | 56,917 | 57,027 | + | 36 |
| *psaJ* | 129 | 64,207 | 64,335 | + | 42 |
| 2. Assembly factors of photosystem I | | | | | |
| *ycf3* | **519** |  |  | - | **172** |
| *ycf3a* | 132 | 43,471 | 43,602 | - | 44 |
| *ycf3b* | 226 | 42,497 | 42,722 | - | 75 |
| *ycf3c* | 161 | 41,613 | 41,773 | - | 53 |
| *ycf4* | 558 | 57,336 | 57,893 | + | 185 |
| 3. Subunits of photosystem II | | | | | |
| *psbA* | 1,062 | 88 | 1,149 | - | 353 |
| *psbB* | 1,527 | 68,408 | 69,934 | + | 508 |
| *psbC* | 1,422 | 9,739 | 11,160 | + | 473 |
| *psbD* | 1,062 | 8,730 | 9,791 | + | 353 |
| *psbE* | 252 | 61,545 | 61,796 | - | 83 |
| *psbF* | 120 | 61,415 | 61,534 | - | 39 |
| *psbH* | 222 | 70,503 | 70,724 | + | 73 |
| *psbI* | 111 | 7,429 | 7,539 | + | 36 |
| *psbJ* | 123 | 61,027 | 61,149 | - | 40 |
| *psbK* | 186 | 6,840 | 7,025 | + | 61 |
| *psbL* | 117 | 61,276 | 61,392 | - | 38 |
| *psbM* | 105 | 16,596 | 16,700 | + | 34 |
| *psbT* | 117 | 70,103 | 70,219 | + | 38 |
| *psbZ* | 189 | 11,758 | 11,946 | + | 62 |
| 4. Subunits of the cytochrome b_6_/f complex | | | | | |
| *petA* | 963 | 59,268 | 60,230 | + | 320 |
| *petB* | **648** |  |  | + | **215** |
| *petBa* | 6 | 70,854 | 70,859 | + | 2 |
| *petBb* | 642 | 71,612 | 72,253 | + | 213 |
| *petD* | **483** |  |  | + | **160** |
| *petDa* | 8 | 72,444 | 72,451 | + | 2 |
| *petDb* | 475 | 73,168 | 73,642 | + | 158 |
| *petG* | 114 | 63,346 | 63,459 | + | 37 |
| *petL* | 96 | 63,077 | 63,172 | + | 31 |
| *petN* | 90 | 16,979 | 17,068 | - | 29 |
| 5. Cytochrome c synthase | | | | | |
| *ccsA* | 960 | 105,524 | 106,483 | + | 319 |
| 6. Photosystem biogenesis factor 1 | | | | | |
| *pbf1* | 132 | 70,268 | 70,399 | - | 43 |
| 7. Subunits of ATP synthase | | | | | |
| *atpA* | 1,524 | 33,932 | 35,455 | + | 507 |
| *atpB* | 1,497 | 52,010 | 53,506 | - | 498 |
| *atpE* | 414 | 51,600 | 52,013 | - | 137 |
| *atpF* | **567** |  |  | + | **188** |
| *atpFa* | 158 | 32,457 | 32,614 | + | 52 |
| *atpFb* | 409 | 33,432 | 33,840 | + | 136 |
| *atpH* | 246 | 31,749 | 31,994 | + | 81 |
| *atpI* | 744 | 30,442 | 31,185 | + | 247 |
| 8. Subunits of NADH-dehydrogenase | | | | | |
| *ndhA* | **1,089** |  |  | - | **362** |
| *ndhAa* | 550 | 112,359 | 112,908 | - | 183 |
| *ndhAb* | 539 | 110,799 | 111,337 | - | 179 |
| *ndhB-1* | **1,533** |  |  | - | **510** |
| *ndhB-1a* | 777 | 87,155 | 87,931 | - | 259 |
| *ndhB-1b* | 756 | 85,684 | 86,439 | - | 251 |
| *ndhB-2* | **1,533** |  |  | + | **510** |
| *ndhB-2a* | 777 | 127,414 | 128,190 | + | 259 |
| *ndhB-2b* | 756 | 128,906 | 129,661 | + | 251 |
| *ndhC* | 363 | 49,341 | 49,703 | - | 120 |
| *ndhD* | 1,509 | 106,626 | 108,134 | - | 502 |
| *ndhE* | 306 | 108,998 | 109,303 | - | 101 |
| *ndhF* | 2,226 | 101,528 | 103,753 | - | 741 |
| *ndhG* | 531 | 109,516 | 110,046 | - | 176 |
| *ndhH* | 1,182 | 112,910 | 114,091 | - | 393 |
| *ndhI* | 543 | 110,162 | 110,704 | - | 180 |
| *ndhJ* | 480 | 48,031 | 48,510 | - | 159 |
| *ndhK* | 741 | 48,610 | 49,350 | - | 246 |
| 9. Large subunit of RubisCo | | | | | |
| *rbcL* | 1,434 | 54,296 | 55,729 | + | 477 |
| 10. Subunits of the DNA-dependent RNA polymerase | | | | | |
| *rpoA* | 1,026 | 73,853 | 74,878 | - | 341 |
| *rpoB* | 3,231 | 19,266 | 22,496 | + | 1076 |
| *rpoC1* | 2,031 | 22,534 | 24,564 | + | 676 |
| *rpoC2* | 4,422 | 24,768 | 29,189 | + | 1473 |
| 11. Small subunits of ribosomal proteins | | | | | |
| *rps2* | 711 | 29,477 | 30,187 | + | 236 |
| *rps3* | 720 | 78,730 | 79,449 | - | 239 |
| *rps4* | 606 | 44,551 | 45,156 | - | 201 |
| *rps7-1* | 471 | 88,231 | 88,701 | - | 156 |
| *rps7-2* | 471 | 126,644 | 127,114 | + | 156 |
| *rps8* | 411 | 76,221 | 76,631 | - | 136 |
| *rps11* | 432 | 74,943 | 75,374 | - | 143 |
| *rps12-1* | **375** |  |  | x | **124** |
| *rps12-1a* | 114 | 66,936 | 67,049 | - | 38 |
| *rps12-1b* | 232 | 89,324 | 89,555 | - | 77 |
| *rps12-1c* | 29 | 88,755 | 88,783 | - | 9 |
| *rps12-2* | **375** |  |  | x | **124** |
| *rps12-2a* | 114 | 66,936 | 67,049 | - | 38 |
| *rps12-2b* | 232 | 125,790 | 126,021 | + | 77 |
| *rps12-2c* | 29 | 126,562 | 126,590 | + | 9 |
| *rps14* | 312 | 36,004 | 36,315 | - | 103 |
| *rps15-1* | 273 | 100,852 | 101,124 | + | 90 |
| *rps15-2* | 273 | 114,221 | 114,493 | - | 90 |
| *rps16* | **270** |  |  | - | **89** |
| *rps16a* | 40 | 5,603 | 5,642 | - | 13 |
| *rps16b* | 230 | 4,531 | 4,760 | - | 76 |
| *rps18* | 450 | 65,267 | 65,716 | + | 149 |
| *rps19-1* | 267 | 80,044 | 80,310 | - | 88 |
| *rps19-2* | 267 | 135,035 | 135,301 | + | 88 |
| 12. Large subunits of ribosomal proteins | | | | | |
| *rpl2-1* | **822** |  |  | - | **273** |
| *rpl2-1a* | 391 | 81,682 | 82,072 | - | 130 |
| *rpl2-1b* | 431 | 80,588 | 81,018 | - | 143 |
| *rpl2-2* | **822** |  |  | + | **273** |
| *rpl2-2a* | 391 | 133,273 | 133,663 | + | 130 |
| *rpl2-2b* | 431 | 134,327 | 134,757 | + | 143 |
| *rpl14* | 372 | 76,775 | 77,146 | - | 123 |
| *rpl16* | **411** |  |  | - | **136** |
| *rpl16a* | 9 | 78,558 | 78,566 | - | 3 |
| *rpl16b* | 402 | 77,268 | 77,669 | - | 133 |
| *rpl20* | 360 | 65,879 | 66,238 | - | 119 |
| *rpl22* | 444 | 79,522 | 79,965 | - | 147 |
| *rpl23-1* | 282 | 82,091 | 82,372 | - | 93 |
| *rpl23-2* | 282 | 132,973 | 133,254 | + | 93 |
| *rpl32* | 180 | 104,524 | 104,703 | + | 59 |
| *rpl33* | 201 | 64,767 | 64,967 | + | 66 |
| *rpl36* | 114 | 75,578 | 75,691 | - | 37 |
| 13. Translational initiation factor I | | | | | |
| *infA* | 342 | 75,797 | 76,138 | - | 113 |
| 14. Other protein coding genes | | | | | |
| Maturase (*matK*) | 1,536 | 1,697 | 3,232 | - | 511 |
| Envelope membrane protein (*cemA*) | 693 | 58,353 | 59,045 | + | 230 |
| Acetyl-coenzyme A carboxylase, carboxyl transferase subunit beta (*accD*) | 153 | 56,480 | 56,632 | + | 50 |
| Clp protease proteolytic subunit (*clpP1*) | 651 | 67,191 | 67,841 | - | 216 |
| 15. Pseudo protein coding genes | | | | | |
| *ndhH-p* | 180 | 101,254 | 101,433 | + | 59 |
| *rpl23-p* | 72 | 56,010 | 56,081 | + | 23 |
| 16. Hypothetical protein coding genes | | | | | |
| *ycf1-1* | 120 | 99,649 | 99,768 | + | 39 |
| *ycf1-2* | 120 | 115,577 | 115,696 | - | 39 |
| *ycf2-1* | 96 | 82,713 | 82,808 | + | 31 |
| *ycf2-2* | 96 | 132,537 | 132,632 | - | 31 |
| *ycf68-1* | 381 | 93,428 | 93,808 | + | 126 |
| *ycf68-2* | 381 | 121,537 | 121,917 | - | 126 |

B.

| rRNA-coding genes^a^ | Length^b^ (bp) | Position in the genome (bp) | | Direction^c^ | Number of amino acids |
| --- | --- | --- | --- | --- | --- |
|  |  | From | To |  |  |
| *rrn4.5-1* | 95 | 98,243 | 98,337 | + | - |
| *rrn4.5-2* | 95 | 117,008 | 117,102 | - | - |
| *rrn5-1* | 121 | 98,565 | 98,685 | + | - |
| *rrn5-2* | 121 | 116,660 | 116,780 | - | - |
| *rrn16-1* | 1,492 | 91,482 | 92,973 | + | - |
| *rrn16-2* | 1,492 | 122,372 | 123,863 | - | - |
| *rrn23-1* | 2,889 | 95,260 | 98,148 | + | - |
| *rrn23-2* | 2,889 | 117,197 | 120,085 | - | - |

| tRNA-coding genes^a^ | Length^b^ (bp) | Position in the genome (bp) | | Direction^c^ | tRNA type | Anti-codon |
| --- | --- | --- | --- | --- | --- | --- |
|  |  | From | To |  |  |  |
| *trnA-1* | **73** |  |  | + | Ala | UGC |
| *trnA-1a* | 38 | 94,231 | 94,268 | + |  |  |
| *trnA-1b* | 35 | 95,080 | 95,114 | + |  |  |
| *trnA-2* | **73** |  |  | - | Ala | UGC |
| *trnA-2a* | 38 | 121,077 | 121,114 | - |  |  |
| *trnA-2b* | 35 | 120,231 | 120,265 | - |  |  |
| *trnC* | 71 | 17,992 | 18,062 | - | Cys | GCA |
| *trnD* | 74 | 16,049 | 16,122 | + | Asp | GUC |
| *trnE* | 73 | 15,468 | 15,540 | + | Glu | UUC |
| *trnF* | 73 | 47,356 | 47,428 | + | Phe | GAA |
| *trnG-1* | 71 | 12,228 | 12,298 | + | Gly | GCC |
| *trnG-2* | **71** |  |  | - | Gly | UCC |
| *trnG-2a* | 23 | 13,668 | 13,690 | - |  |  |
| *trnG-2b* | 48 | 12,942 | 12,989 | - |  |  |
| *trnH-1* | 75 | 80,458 | 80,532 | + | His | GUG |
| *trnH-2* | 75 | 134,813 | 134,887 | - | His | GUG |
| *trnI-1* | 74 | 82,547 | 82,620 | - | Ile | CAU |
| *trnI-2* | **77** |  |  | + | Ile | GAU |
| *trnI-2a* | 42 | 93,287 | 93,328 | + |  |  |
| *trnI-2b* | 35 | 94,131 | 94,165 | + |  |  |
| *trnI-3* | **77** |  |  | - | Ile | GAU |
| *trnI-3a* | 42 | 122,017 | 122,058 | - |  |  |
| *trnI-3b* | 35 | 121,180 | 121,214 | - |  |  |
| *trnI-4* | 74 | 132,725 | 132,798 | + | Ile | CAU |
| *trnK* | **72** |  |  | - | Lys | UUU |
| *trnKa* | 37 | 3,916 | 3,952 | - |  |  |
| *trnKb* | 35 | 1,384 | 1,418 | - |  |  |
| *trnL-1* | **85** |  |  | + | Leu | UAA |
| *trnL-1a* | 35 | 46,380 | 46,414 | + |  |  |
| *trnL-1b* | 50 | 46,965 | 47,014 | + |  |  |
| *trnL-2* | 81 | 85,071 | 85,151 | - | Leu | CAA |
| *trnL-3* | 80 | 105,367 | 105,446 | + | Leu | UAG |
| *trnL-4* | 81 | 130,194 | 130,274 | + | Leu | CAA |
| *trnfM* | 74 | 12,778 | 12,851 | - | Met | CAU |
| *trnM-1* | 59 | 14,864 | 14,922 | + | Met | CAU |
| *trnM-2* | 71 | 45,477 | 45,547 | - | Met | CAU |
| *trnM-3* | 73 | 51,413 | 51,485 | + | Met | CAU |
| *trnN-1* | 73 | 99,240 | 99,312 | - | Asn | GUU |
| *trnN-2* | 73 | 116,033 | 116,105 | + | Asn | GUU |
| *trnP* | 75 | 63,796 | 63,870 | - | Pro | UGG |
| *trnQ* | 73 | 6,424 | 6,496 | - | Gln | UUG |
| *trnR-1* | 72 | 35,553 | 35,624 | - | Arg | UCU |
| *trnR-2* | 74 | 98,914 | 98,987 | + | Arg | ACG |
| *trnR-3* | 74 | 116,358 | 116,431 | - | Arg | ACG |
| *trnS-1* | 88 | 7,658 | 7,745 | - | Ser | GCU |
| *trnS-2* | 88 | 11,316 | 11,403 | - | Ser | UGA |
| *trnS-3* | 87 | 44,199 | 44,285 | + | Ser | GGA |
| *trnT-1* | 72 | 14,858 | 14,929 | + | Thr | GGU |
| *trnT-2* | 61 | 14,930 | 14,990 | + | Thr | GGU |
| *trnT-3* | 73 | 45,475 | 45,547 | - | Thr | UGU |
| *trnV-1* | **76** |  |  | - | Val | UAC |
| *trnV-1a* | 39 | 51,188 | 51,226 | - |  |  |
| *trnV-1b* | 37 | 50,548 | 50,584 | - |  |  |
| *trnV-2* | 72 | 91,181 | 91,252 | + | Val | GAC |
| *trnV-3* | 72 | 124,093 | 124,164 | - | Val | GAC |
| *trnW* | 74 | 63,582 | 63,655 | - | Trp | CCA |
| *trnY* | 84 | 15,602 | 15,685 | + | Tyr | GUA |

^a^p, pseudogene; ^b^Boldface, sum of all exons; lower-case letters, exon of genes; hyphenated, duplicate genes; ^c^Plus and minus, forward and reverse DNA strand, respectively; x, trans-spliced.
