# Supplementary material for: Dig up tall fescue plastid genomes for the identification of morphotype-specific DNA variants
Source: BMC Genomics. 2023 Oct 3;24:586. doi: 10.1186/s12864-023-09631-8 (PMC10546690; doi:10.1186/s12864-023-09631-8)
Supplement: Supplementary file 1 — Additional file 1: Tables S1-S13 [file 12864_2023_9631_MOESM1_ESM.zip › Additional file 1 Table S3_updated_ESM.docx]

**Additional file 1: Table S3.** Genes identified in Mediterranean cv. Resolute tall fescue plastid genome.

A.

| Protein-coding genes^a^ | Length^b^ (bp) | Position in the genome (bp) | | Direction^c^ | Number of amino acids |
| --- | --- | --- | --- | --- | --- |
|  |  | From | To |  |  |
| 1. Subunits of photosystem I | | | | | |
| *psaA* | 2,253 | 38,698 | 40,950 | - | 750 |
| *psaB* | 2,205 | 36,468 | 38,672 | - | 734 |
| *psaC* | 246 | 108,213 | 108,458 | - | 81 |
| *psaI* | 111 | 56,886 | 56,996 | + | 36 |
| *psaJ* | 129 | 64,171 | 64,299 | + | 42 |
| 2. Assembly factors of photosystem I | | | | | |
| *ycf3* | **519** |  |  | - | **172** |
| *ycf3a* | 132 | 43,465 | 43,596 | - | 44 |
| *ycf3b* | 226 | 42,491 | 42,716 | - | 75 |
| *ycf3c* | 161 | 41,602 | 41,762 | - | 53 |
| *ycf4* | 558 | 57,305 | 57,862 | + | 185 |
| 3. Subunits of photosystem II | | | | | |
| *psbA* | 1,062 | 88 | 1,149 | - | 353 |
| *psbB* | 1,527 | 68,388 | 69,914 | + | 508 |
| *psbC* | 1,422 | 9,743 | 11,164 | + | 473 |
| *psbD* | 1,062 | 8,734 | 9,795 | + | 353 |
| *psbE* | 252 | 61,509 | 61,760 | - | 83 |
| *psbF* | 120 | 61,379 | 61,498 | - | 39 |
| *psbH* | 222 | 70,483 | 70,704 | + | 73 |
| *psbI* | 111 | 7,431 | 7,541 | + | 36 |
| *psbJ* | 123 | 60,991 | 61,113 | - | 40 |
| *psbK* | 186 | 6,840 | 7,025 | + | 61 |
| *psbL* | 117 | 61,240 | 61,356 | - | 38 |
| *psbM* | 105 | 16,585 | 16,689 | + | 34 |
| *psbT* | 117 | 70,083 | 70,199 | + | 38 |
| *psbZ* | 189 | 11,761 | 11,949 | + | 62 |
| 4. Subunits of the cytochrome b_6_/f complex | | | | | |
| *petA* | 963 | 59,238 | 60,200 | + | 320 |
| *petB* | **648** |  |  | + | **215** |
| *petBa* | 6 | 70,834 | 70,839 | + | 2 |
| *petBb* | 642 | 71,597 | 72,238 | + | 213 |
| *petD* | **483** |  |  | + | **160** |
| *petDa* | 8 | 72,429 | 72,436 | + | 2 |
| *petDb* | 475 | 73,120 | 73,594 | + | 158 |
| *petG* | 114 | 63,310 | 63,423 | + | 37 |
| *petL* | 96 | 63,041 | 63,136 | + | 31 |
| *petN* | 90 | 16,968 | 17,057 | - | 29 |
| 5. Cytochrome c synthase | | | | | |
| *ccsA* | 966 | 105,473 | 106,438 | + | 321 |
| 6. Photosystem biogenesis factor 1 | | | | | |
| *pbf1* | 132 | 70,248 | 70,379 | - | 43 |
| 7. Subunits of ATP synthase | | | | | |
| *atpA* | 1,524 | 33,890 | 35,413 | + | 507 |
| *atpB* | 1,497 | 51,974 | 53,470 | - | 498 |
| *atpE* | 414 | 51,564 | 51,977 | - | 137 |
| *atpF* | **567** |  |  | + | **188** |
| *atpFa* | 158 | 32,423 | 32,580 | + | 52 |
| *atpFb* | 409 | 33,390 | 33,798 | + | 136 |
| *atpH* | 246 | 31,715 | 31,960 | + | 81 |
| *atpI* | 744 | 30,409 | 31,152 | + | 247 |
| 8. Subunits of NADH-dehydrogenase | | | | | |
| *ndhA* | **1,089** |  |  | - | **362** |
| *ndhAa* | 548 | 112,350 | 112,897 | - | 182 |
| *ndhAb* | 541 | 110,792 | 111,332 | - | 180 |
| *ndhB-1* | **1,533** |  |  |  | **510** |
| *ndhB-1a* | 777 | 87,145 | 87,921 | - | 259 |
| *ndhB-1b* | 756 | 85,674 | 86,429 | - | 251 |
| *ndhB-2* | **1,533** |  |  |  | **510** |
| *ndhB-2a* | 777 | 127,401 | 128,177 | + | 259 |
| *ndhB-2b* | 756 | 128,893 | 129,648 | + | 251 |
| *ndhC* | 363 | 49,308 | 49,670 | - | 120 |
| *ndhD* | 1,509 | 106,585 | 108,093 | - | 502 |
| *ndhE* | 306 | 108,957 | 109,262 | - | 101 |
| *ndhF* | 2,226 | 101,538 | 103,763 | - | 741 |
| *ndhG* | 531 | 109,475 | 110,005 | - | 176 |
| *ndhH* | 1,182 | 112,899 | 114,080 | - | 393 |
| *ndhI* | 543 | 110,124 | 110,666 | - | 180 |
| *ndhJ* | 480 | 47,998 | 48,477 | - | 159 |
| *ndhK* | 741 | 48,577 | 49,317 | - | 246 |
| 9. Large subunit of RubisCo | | | | | |
| *rbcL* | 1434 | 54,260 | 55,693 | + | 477 |
| 10. Subunits of the DNA-dependent RNA polymerase | | | | | |
| *rpoA* | 1,026 | 73,805 | 74,830 | - | 341 |
| *rpoB* | 3,231 | 19,225 | 22,455 | + | 1076 |
| *rpoC1* | 2,031 | 22,493 | 24,523 | + | 676 |
| *rpoC2* | 4,422 | 24,727 | 29,148 | + | 1473 |
| 11. Small subunits of ribosomal proteins | | | | | |
| *rps2* | 711 | 29,444 | 30,154 | + | 236 |
| *rps3* | 720 | 78,671 | 79,390 | - | 239 |
| *rps4* | 606 | 44,564 | 45,169 | - | 201 |
| *rps7-1* | 471 | 88,221 | 88,691 | - | 156 |
| *rps7-2* | 471 | 126,631 | 127,101 | + | 156 |
| *rps8* | 411 | 76,163 | 76,573 | - | 136 |
| *rps11* | 432 | 74,895 | 75,326 | - | 143 |
| *rps12-1* | **375** |  |  | x | **124** |
| *rps12-1a* | 114 | 66,939 | 67,052 | - | 38 |
| *rps12-1b* | 232 | 89,314 | 89,545 | - | 77 |
| *rps12-1c* | 29 | 88,745 | 88,773 | - | 9 |
| *rps12-2* | **375** |  |  | x | **124** |
| *rps12-2a* | 114 | 66,939 | 67,052 | - | 38 |
| *rps12-2b* | 232 | 125,777 | 126,008 | + | 77 |
| *rps12-2c* | 29 | 126,549 | 126,577 | + | 9 |
| *rps14* | 312 | 35,996 | 36,307 | - | 103 |
| *rps15-1* | 273 | 100,840 | 101,112 | + | 90 |
| *rps15-2* | 273 | 114,210 | 114,482 | - | 90 |
| *rps16* | **270** | - | - | - | **89** |
| *rps16a* | 40 | 5,608 | 5,647 | - | 13 |
| *rps16b* | 230 | 4,536 | 4,765 | - | 76 |
| *rps18* | 471 | 65,245 | 65,715 | + | 156 |
| *rps19-1* | 267 | 79,993 | 80,259 | - | 88 |
| *rps19-2* | 267 | 135,016 | 135,282 | + | 88 |
| 12. Large subunits of ribosomal proteins | | | | | |
| *rpl2-1* | **822** |  |  | - | **273** |
| *rpl2-1a* | 391 | 81,630 | 82,020 | - | 130 |
| *rpl2-1b* | 431 | 80,536 | 80,966 | - | 143 |
| *rpl2-2* | **822** |  |  | + | **273** |
| *rpl2-2a* | 391 | 133,255 | 133,645 | + | 130 |
| *rpl2-2b* | 431 | 134,309 | 134,739 | + | 143 |
| *rpl14* | 372 | 76,721 | 77,092 | - | 123 |
| *rpl16* | **411** |  |  |  | **136** |
| *rpl16a* | 9 | 78,498 | 78,506 | - | 3 |
| *rpl16b* | 402 | 77,221 | 77,622 | - | 133 |
| *rpl20* | 360 | 65,878 | 66,237 | - | 119 |
| *rpl22* | 444 | 79,464 | 79,907 | - | 147 |
| *rpl23-1* | 282 | 82,039 | 82,320 | - | 93 |
| *rpl23-2* | 282 | 132,955 | 133,236 | + | 93 |
| *rpl32* | 180 | 104,483 | 104,662 | + | 59 |
| *rpl33* | 201 | 64,732 | 64,932 | + | 66 |
| *rpl36* | 114 | 75,520 | 75,633 | - | 37 |
| 13. Translational initiation factor I | | | | | |
| *infA* | 342 | 75,739 | 76,080 | - | 113 |
| 14. Other protein coding genes | | | | | |
| Maturase (*matK*) | 1,536 | 1,696 | 3,231 | - | 511 |
| Envelope membrane protein (*cemA*) | 693 | 58,324 | 59,016 | + | 230 |
| Acetyl-coenzyme A carboxylase, carboxyl transferase subunit beta (*accD*) | 165 | 56,449 | 56,613 | + | 54 |
| Clp protease proteolytic subunit (*clpP1*) | 651 | 67,194 | 67,844 | - | 216 |
| 15. Pseudo protein coding genes | | | | | |
| *ndhH-p* | 189 | 101,242 | 101,430 | + | 62 |
| *rpl23-p* | 72 | 55,974 | 56,045 | + | 23 |
| 16. Hypothetical protein coding genes | | | | | |
| *ycf1-1* | 120 | 99,639 | 99,758 | + | 39 |
| *ycf1-2* | 120 | 115,564 | 115,683 | - | 39 |
| *ycf2-1* | 96 | 82,661 | 82,756 | + | 31 |
| *ycf2-2* | 96 | 132,519 | 132,614 | - | 31 |
| *ycf68-1* | 381 | 93,418 | 93,798 | + | 126 |
| *ycf68-2* | 381 | 121,524 | 121,904 | - | 126 |

B.

| rRNA-coding genes^a^ | Length^b^ (bp) | Position in the genome (bp) | | Direction^c^ | Number of amino acids |
| --- | --- | --- | --- | --- | --- |
|  |  | From | To |  |  |
| *rrn4.5-1* | 95 | 98,233 | 98,327 | + | - |
| *rrn4.5-2* | 95 | 116,995 | 117,089 | - | - |
| *rrn5-1* | 121 | 98,555 | 98,675 | + | - |
| *rrn5-2* | 121 | 116,647 | 116,767 | - | - |
| *rrn16-1* | 1,492 | 91,472 | 92,963 | + | - |
| *rrn16-2* | 1,492 | 122,359 | 123,850 | - | - |
| *rrn23-1* | 2,889 | 95,250 | 98,138 | + | - |
| *rrn23-2* | 2,889 | 117,184 | 120,072 | - | - |

| tRNA-coding genes^a^ | Length^b^ (bp) | Position in the genome (bp) | | Direction^c^ | tRNA type | Anti-codon |
| --- | --- | --- | --- | --- | --- | --- |
|  |  | From | To |  |  |  |
| *trnA-1* | **73** |  |  | + | Ala | UGC |
| *trnA-1a* | 38 | 94,221 | 94,258 | + |  |  |
| *trnA-1b* | 35 | 95,070 | 95,104 | + |  |  |
| *trnA-2* | **73** |  |  | - | Ala | UGC |
| *trnA-2a* | 38 | 121,064 | 121,101 | - |  |  |
| *trnA-2b* | 35 | 120,218 | 120,252 | - |  |  |
| *trnC* | 71 | 17,978 | 18,048 | - | Cys | GCA |
| *trnD* | 74 | 16,037 | 16,110 | + | Asp | GUC |
| *trnE* | 73 | 15,456 | 15,528 | + | Glu | UUC |
| *trnF* | 73 | 47,343 | 47,415 | + | Phe | GAA |
| *trnG-1* | 71 | 12,232 | 12,302 | + | Gly | GCC |
| *trnG-2* | **71** |  |  | - | Gly | UCC |
| *trnG-2a* | 23 | 13,668 | 13,690 | - |  |  |
| *trnG-2b* | 48 | 12,943 | 12,990 | - |  |  |
| *trnH-1* | 75 | 80,406 | 80,480 | + | His | GUG |
| *trnH-2* | 75 | 134,795 | 134,869 | - | His | GUG |
| *trnI-1* | 74 | 82,495 | 82,568 | - | Ile | CAU |
| *trnI-2* | **77** |  |  | + | Ile | GAU |
| *trnI-2a* | 42 | 93,277 | 93,318 | + |  |  |
| *trnI-2b* | 35 | 94,121 | 94,155 | + |  |  |
| *trnI-3* | **77** |  |  | - | Ile | GAU |
| *trnI-3a* | 42 | 122,004 | 122,045 | - |  |  |
| *trnI-3b* | 35 | 121,167 | 121,201 | - |  |  |
| *trnI-4* | 74 | 132,707 | 132,780 | + | Ile | CAU |
| *trnK* | **72** | - | - | - | Lys | UUU |
| *trnKa* | 37 | 3,918 | 3,954 | - |  |  |
| *trnKb* | 35 | 1,384 | 1,418 | - |  |  |
| *trnL-1* | **85** |  |  | + | Leu | UAA |
| *trnL-1a* | 35 | 46,372 | 46,406 | + |  |  |
| *trnL-1b* | 50 | 46,951 | 47,000 | + |  |  |
| *trnL-2* | 81 | 85,061 | 85,141 | - | Leu | CAA |
| *trnL-3* | 80 | 105,316 | 105,395 | + | Leu | UAG |
| *trnL-4* | 81 | 130,181 | 130,261 | + | Leu | CAA |
| *trnfM* | 74 | 12,787 | 12,860 | - | Met | CAU |
| *trnM-1* | 59 | 14,862 | 14,920 | + | Met | CAU |
| *trnM-2* | 71 | 45,476 | 45,546 | - | Met | CAU |
| *trnM-3* | 73 | 51,377 | 51,449 | + | Met | CAU |
| *trnN-1* | 73 | 99,230 | 99,302 | - | Asn | GUU |
| *trnN-2* | 73 | 116,020 | 116,092 | + | Asn | GUU |
| *trnP* | 75 | 63,760 | 63,834 | - | Pro | UGG |
| *trnQ* | 73 | 6,424 | 6,496 | - | Gln | UUG |
| *trnR-1* | 72 | 35,545 | 35,616 | - | Arg | UCU |
| *trnR-2* | 74 | 98,904 | 98,977 | + | Arg | ACG |
| *trnR-3* | 74 | 116,345 | 116,418 | - | Arg | ACG |
| *trnS-1* | 88 | 7,661 | 7,748 | - | Ser | GCU |
| *trnS-2* | 88 | 11,320 | 11,407 | - | Ser | UGA |
| *trnS-3* | 87 | 44,211 | 44,297 | + | Ser | GGA |
| *trnT-1* | 72 | 14,856 | 14,927 | + | Thr | GGU |
| *trnT-2* | 61 | 14,928 | 14,988 | + | Thr | GGU |
| *trnT-3* | 73 | 45,474 | 45,546 | - | Thr | UGU |
| *trnV-1* | **76** |  |  | - | Val | UAC |
| *trnV-1a* | 39 | 51,152 | 51,190 | - |  |  |
| *trnV-1b* | 37 | 50,512 | 50,548 | - |  |  |
| *trnV-2* | 72 | 91,171 | 91,242 | + | Val | GAC |
| *trnV-3* | 72 | 124,080 | 124,151 | - | Val | GAC |
| *trnW* | 74 | 63,546 | 63,619 | - | Trp | CCA |
| *trnY* | 84 | 15,590 | 15,673 | + | Tyr | GUA |

^a^p, pseudogene; ^b^Boldface, sum of all exons; lower-case letters, exon of genes; hyphenated, duplicate genes; ^c^Plus and minus, forward and reverse DNA strand, respectively; x, trans-spliced.
