# Supplementary material for: Dig up tall fescue plastid genomes for the identification of morphotype-specific DNA variants
Source: BMC Genomics. 2023 Oct 3;24:586. doi: 10.1186/s12864-023-09631-8 (PMC10546690; doi:10.1186/s12864-023-09631-8)
Supplement: Supplementary file 1 — Additional file 1: Tables S1-S13 [file 12864_2023_9631_MOESM1_ESM.zip › Additional file 1 Table S4_updated_ESM.docx]

**Additional file 1: Table S4**. Tandem repeat identified in Continental cv. Texoma MaxQ II tall fescue plastid genome.

| Indices | | Period size | Copy number | Consensus size | Percent matches | Percent Indels | Alignment score |
| --- | --- | --- | --- | --- | --- | --- | --- |
| From | To |  |  |  |  |  |  |
| 12333 | 12394 | 29 | 2.1 | 30 | 93 | 3 | 108 |
| 12512 | 12563 | 25 | 2.1 | 25 | 92 | 0 | 86 |
| 24694 | 24734 | 21 | 2 | 21 | 100 | 0 | 82 |
| 26584 | 26704 | 48 | 2.4 | 48 | 83 | 8 | 134 |
| 26676 | 26746 | 21 | 3.4 | 20 | 86 | 11 | 90 |
| 26628 | 26724 | 48 | 2 | 48 | 86 | 7 | 135 |
| 26578 | 26918 | 75 | 4.8 | 74 | 74 | 13 | 252 |
| 44020 | 44066 | 21 | 2.2 | 21 | 96 | 0 | 85 |
| 65269 | 65372 | 21 | 5 | 21 | 84 | 4 | 138 |
| 67958 | 68032 | 24 | 3.1 | 24 | 96 | 0 | 132 |
| 75762 | 75805 | 18 | 2.4 | 18 | 100 | 0 | 88 |
| 75762 | 75830 | 36 | 1.9 | 36 | 82 | 5 | 86 |
| 78339 | 78393 | 27 | 2 | 27 | 100 | 0 | 110 |
| 85386 | 85439 | 27 | 2 | 27 | 100 | 0 | 108 |
| 129798 | 129851 | 27 | 2 | 27 | 100 | 0 | 108 |
| Average | | 31.2 | 2.62 |  |  |  |  |
