# Supplementary material for: Dig up tall fescue plastid genomes for the identification of morphotype-specific DNA variants
Source: BMC Genomics. 2023 Oct 3;24:586. doi: 10.1186/s12864-023-09631-8 (PMC10546690; doi:10.1186/s12864-023-09631-8)
Supplement: Supplementary file 1 — Additional file 1: Tables S1-S13 [file 12864_2023_9631_MOESM1_ESM.zip › Additional file 1 Table S6_updated_ESM.docx]

**Additional file 1: Table S6**. Tandem repeat identified in Mediterranean cv. Resolute tall fescue plastid genome.

| Indices | | Period  size | Copy  number | Consensus  size | Percent matches | Percent  Indels | Alignment score |
| --- | --- | --- | --- | --- | --- | --- | --- |
| From | To |  |  |  |  |  |  |
| 12341 | 12402 | 29 | 2.1 | 30 | 93 | 3 | 108 |
| 24666 | 24706 | 21 | 2 | 21 | 100 | 0 | 82 |
| 26600 | 26696 | 48 | 2 | 48 | 86 | 7 | 135 |
| 26550 | 26831 | 75 | 3.8 | 75 | 73 | 13 | 203 |
| 65253 | 65377 | 21 | 6 | 21 | 85 | 3 | 144 |
| 67962 | 68019 | 24 | 2.5 | 24 | 88 | 5 | 84 |
| 75759 | 75802 | 18 | 2.4 | 18 | 100 | 0 | 88 |
| 75759 | 75827 | 36 | 1.9 | 36 | 82 | 5 | 86 |
| 84931 | 85025 | 47 | 2 | 47 | 100 | 0 | 190 |
| 85431 | 85484 | 27 | 2 | 27 | 100 | 0 | 108 |
| 101404 | 101465 | 31 | 2 | 31 | 100 | 0 | 124 |
| 110739 | 110798 | 30 | 2 | 29 | 96 | 3 | 111 |
| 129838 | 129891 | 27 | 2 | 27 | 100 | 0 | 108 |
| Average | | 33.38 | 2.52 |  |  |  |  |
