# Supplementary material for: Dig up tall fescue plastid genomes for the identification of morphotype-specific DNA variants
Source: BMC Genomics. 2023 Oct 3;24:586. doi: 10.1186/s12864-023-09631-8 (PMC10546690; doi:10.1186/s12864-023-09631-8)
Supplement: Supplementary file 1 — Additional file 1: Tables S1-S13 [file 12864_2023_9631_MOESM1_ESM.zip › Additional file 1 Table S7_updated_ESM.docx]

**Additional file 1: Table S7**. List of simple sequence repeats (SSRs) identified in Continental cv. Texoma MaxQ II tall fescue plastid genome.

| **SSR number** | **SSR type** | **SSR** | **Size** | **Start (bp)** | **End (bp)** |
| --- | --- | --- | --- | --- | --- |
| 1 | Trinucleotide | (AAG)3 | 9 | 326 | 334 |
| 2 | Trinucleotide | (AAT)3 | 9 | 3551 | 3559 |
| 3 | Trinucleotide | (TCT)3 | 9 | 4507 | 4515 |
| 4 | Trinucleotide | (TTA)3 | 9 | 5891 | 5899 |
| 5 | Mononucleotide | (A)10 | 10 | 6053 | 6062 |
| 6 | Mononucleotide | (T)11 | 11 | 7299 | 7309 |
| 7 | Mononucleotide | (T)11 | 11 | 7808 | 7818 |
| 8 | Mononucleotide | (T)12 | 12 | 8352 | 8363 |
| 9 | Tetranucleotide | (ATAC)3 | 12 | 16243 | 16254 |
| 10 | Mononucleotide | (T)11 | 11 | 17275 | 17285 |
| 11 | Mononucleotide | (A)10 | 10 | 17698 | 17707 |
| 12 | Pentanucleotide | (TATTA)3 | 15 | 18887 | 18901 |
| 13 | Mononucleotide | (A)13 | 13 | 20653 | 20665 |
| 14 | Trinucleotide | (AAC)3 | 9 | 23427 | 23435 |
| 15 | Trinucleotide | (TAT)3 | 9 | 25010 | 25018 |
| 16 | Trinucleotide | (AGA)3 | 9 | 26611 | 26619 |
| 17 | Trinucleotide | (AAG)3 | 9 | 27441 | 27449 |
| 18 | Trinucleotide | (ATT)3 | 9 | 29343 | 29351 |
| 19 | Mononucleotide | (A)14 | 14 | 29425 | 29438 |
| 20 | Trinucleotide | (GTT)4 | 12 | 31029 | 31040 |
| 21 | Mononucleotide | (T)12 | 12 | 31198 | 31209 |
| 22 | Trinucleotide | (TGC)3 | 9 | 31754 | 31762 |
| 23 | Trinucleotide | (TCT)3 | 9 | 36270 | 36278 |
| 24 | Trinucleotide | (TTG)3 | 9 | 39446 | 39454 |
| 25 | Trinucleotide | (GCA)3 | 9 | 39739 | 39747 |
| 26 | Mononucleotide | (T)10 | 10 | 42221 | 42230 |
| 27 | Trinucleotide | (AGT)3 | 9 | 42306 | 42314 |
| 28 | Tetranucleotide | (AAAC)4 | 16 | 43649 | 43664 |
| 29 | Trinucleotide | (CAA)3 | 9 | 43847 | 43855 |
| 30 | Mononucleotide | (A)13 | 13 | 45441 | 45453 |
| 31 | Mononucleotide | (A)12 | 12 | 46326 | 46337 |
| 32 | Mononucleotide | (T)10 | 10 | 47167 | 47176 |
| 33 | Mononucleotide | (A)11 | 11 | 47444 | 47454 |
| 34 | Trinucleotide | (AAC)3 | 9 | 49149 | 49157 |
| 35 | Mononucleotide | (T)12 | 12 | 49309 | 49320 |
| 36 | Trinucleotide | (ATA)3 | 9 | 50040 | 50048 |
| 37 | Mononucleotide | (A)12 | 12 | 50518 | 50529 |
| 38 | Trinucleotide | (TCC)3 | 9 | 52246 | 52254 |
| 39 | Trinucleotide | (AAC)3 | 9 | 54026 | 54034 |
| 40 | Trinucleotide | (GAA)3 | 9 | 57930 | 57938 |
| 41 | Mononucleotide | (T)10 | 10 | 58712 | 58721 |
| 42 | Trinucleotide | (TTC)3 | 9 | 60132 | 60140 |
| 43 | Mononucleotide | (A)10 | 10 | 60737 | 60746 |
| 44 | Mononucleotide | (T)11 | 11 | 62773 | 62783 |
| 45 | Mononucleotide | (A)14 | 14 | 63043 | 63056 |
| 46 | Trinucleotide | (TTC)4 | 12 | 64689 | 64700 |
| 47 | Trinucleotide | (AAC)3 | 9 | 65490 | 65498 |
| 48 | Tetranucleotide | (AGAA)3 | 12 | 67904 | 67915 |
| 49 | Trinucleotide | (GAT)3 | 9 | 73056 | 73064 |
| 50 | Trinucleotide | (AAG)3 | 9 | 73779 | 73787 |
| 51 | Trinucleotide | (TAT)3 | 9 | 74844 | 74852 |
| 52 | Mononucleotide | (T)10 | 10 | 76049 | 76058 |
| 53 | Mononucleotide | (T)10 | 10 | 76067 | 76076 |
| 54 | Mononucleotide | (A)10 | 10 | 78480 | 78489 |
| 55 | Tetranucleotide | (ATTT)3 | 12 | 79461 | 79472 |
| 56 | Trinucleotide | (TTC)3 | 9 | 79861 | 79869 |
| 57 | Trinucleotide | (TTC)3 | 9 | 80586 | 80594 |
| 58 | Trinucleotide | (AGA)3 | 9 | 86266 | 86274 |
| 59 | Trinucleotide | (AGA)3 | 9 | 87740 | 87748 |
| 60 | Trinucleotide | (AAG)3 | 9 | 90765 | 90773 |
| 61 | Trinucleotide | (AAC)3 | 9 | 91559 | 91567 |
| 62 | Tetranucleotide | (AACG)3 | 12 | 98263 | 98274 |
| 63 | Trinucleotide | (CCT)3 | 9 | 101543 | 101551 |
| 64 | Trinucleotide | (TTG)3 | 9 | 102210 | 102218 |
| 65 | Trinucleotide | (TAA)3 | 9 | 103126 | 103134 |
| 66 | Mononucleotide | (A)11 | 11 | 103904 | 103914 |
| 67 | Tetranucleotide | (AACA)3 | 12 | 104633 | 104644 |
| 68 | Tetranucleotide | (ATTA)5 | 20 | 104719 | 104738 |
| 69 | Tetranucleotide | (AATA)3 | 12 | 106774 | 106785 |
| 70 | Trinucleotide | (TTA)3 | 9 | 111978 | 111986 |
| 71 | Trinucleotide | (AGC)3 | 9 | 112361 | 112369 |
| 72 | Tetranucleotide | (TCGT)3 | 12 | 116962 | 116973 |
| 73 | Trinucleotide | (GTT)3 | 9 | 123670 | 123678 |
| 74 | Trinucleotide | (CTT)3 | 9 | 124464 | 124472 |
| 75 | Trinucleotide | (TTC)3 | 9 | 127488 | 127496 |
| 76 | Trinucleotide | (TCT)3 | 9 | 128963 | 128971 |
| 77 | Trinucleotide | (GAA)3 | 9 | 134643 | 134651 |
