# Supplementary material for: Dig up tall fescue plastid genomes for the identification of morphotype-specific DNA variants
Source: BMC Genomics. 2023 Oct 3;24:586. doi: 10.1186/s12864-023-09631-8 (PMC10546690; doi:10.1186/s12864-023-09631-8)
Supplement: Supplementary file 1 — Additional file 1: Tables S1-S13 [file 12864_2023_9631_MOESM1_ESM.zip › Additional file 1 Table S8_updated_ESM.docx]

**Additional file 1: Table S8**. List of simple sequence repeats (SSRs) identified in Rhizomatous cv. Torpedo tall fescue plastid genome.

| **SSR number** | **SSR type** | **SSR** | **Size** | **Start (bp)** | **End (bp)** |
| --- | --- | --- | --- | --- | --- |
| 1 | Trinucleotide | (AAG)3 | 9 | 326 | 334 |
| 2 | Trinucleotide | (AAT)3 | 9 | 3551 | 3559 |
| 3 | Trinucleotide | (TCT)3 | 9 | 4507 | 4515 |
| 4 | Trinucleotide | (TTA)3 | 9 | 5891 | 5899 |
| 5 | Mononucleotide | (A)10 | 10 | 6053 | 6062 |
| 6 | Mononucleotide | (T)12 | 12 | 7299 | 7310 |
| 7 | Mononucleotide | (T)11 | 11 | 7809 | 7819 |
| 8 | Mononucleotide | (T)11 | 11 | 8352 | 8362 |
| 9 | Tetranucleotide | (ATAC)3 | 12 | 16242 | 16253 |
| 10 | Mononucleotide | (T)10 | 10 | 17274 | 17283 |
| 11 | Mononucleotide | (A)10 | 10 | 17696 | 17705 |
| 12 | Mononucleotide | (A)10 | 10 | 18167 | 18176 |
| 13 | Pentanucleotide | (TATTA)3 | 15 | 18877 | 18891 |
| 14 | Mononucleotide | (A)13 | 13 | 20666 | 20678 |
| 15 | Trinucleotide | (AAC)3 | 9 | 23440 | 23448 |
| 16 | Trinucleotide | (TAT)3 | 9 | 25023 | 25031 |
| 17 | Trinucleotide | (AGA)3 | 9 | 26624 | 26632 |
| 18 | Trinucleotide | (AAG)3 | 9 | 27454 | 27462 |
| 19 | Trinucleotide | (ATT)3 | 9 | 29356 | 29364 |
| 20 | Mononucleotide | (A)11 | 11 | 29438 | 29448 |
| 21 | Trinucleotide | (GTT)4 | 12 | 31039 | 31050 |
| 22 | Mononucleotide | (T)11 | 11 | 31208 | 31218 |
| 23 | Trinucleotide | (TGC)3 | 9 | 31763 | 31771 |
| 24 | Trinucleotide | (TCT)3 | 9 | 36279 | 36287 |
| 25 | Trinucleotide | (TTG)3 | 9 | 39455 | 39463 |
| 26 | Trinucleotide | (GCA)3 | 9 | 39748 | 39756 |
| 27 | Mononucleotide | (T)10 | 10 | 42230 | 42239 |
| 28 | Trinucleotide | (AGT)3 | 9 | 42315 | 42323 |
| 29 | Tetranucleotide | (AAAC)4 | 16 | 43658 | 43673 |
| 30 | Trinucleotide | (CAA)3 | 9 | 43856 | 43864 |
| 31 | Mononucleotide | (A)13 | 13 | 45429 | 45441 |
| 32 | Mononucleotide | (A)12 | 12 | 46314 | 46325 |
| 33 | Mononucleotide | (T)10 | 10 | 47155 | 47164 |
| 34 | Trinucleotide | (AAC)3 | 9 | 49155 | 49163 |
| 35 | Mononucleotide | (T)12 | 12 | 49315 | 49326 |
| 36 | Trinucleotide | (ATA)3 | 9 | 50046 | 50054 |
| 37 | Mononucleotide | (A)11 | 11 | 50530 | 50540 |
| 38 | Trinucleotide | (TCC)3 | 9 | 52257 | 52265 |
| 39 | Trinucleotide | (AAC)3 | 9 | 54032 | 54040 |
| 40 | Trinucleotide | (GAA)3 | 9 | 57936 | 57944 |
| 41 | Mononucleotide | (T)10 | 10 | 58718 | 58727 |
| 42 | Trinucleotide | (TTC)3 | 9 | 60138 | 60146 |
| 43 | Mononucleotide | (A)10 | 10 | 60743 | 60752 |
| 44 | Mononucleotide | (T)10 | 10 | 62779 | 62788 |
| 45 | Mononucleotide | (A)14 | 14 | 63048 | 63061 |
| 46 | Mononucleotide | (T)10 | 10 | 64639 | 64648 |
| 47 | Trinucleotide | (TTC)4 | 12 | 64694 | 64705 |
| 48 | Mononucleotide | (T)10 | 10 | 65234 | 65243 |
| 49 | Trinucleotide | (AAC)3 | 9 | 65496 | 65504 |
| 50 | Tetranucleotide | (AGAA)3 | 12 | 67910 | 67921 |
| 51 | Trinucleotide | (GAT)3 | 9 | 73101 | 73109 |
| 52 | Trinucleotide | (AAG)3 | 9 | 73824 | 73832 |
| 53 | Trinucleotide | (TAT)3 | 9 | 74889 | 74897 |
| 54 | Mononucleotide | (T)10 | 10 | 76104 | 76113 |
| 55 | Mononucleotide | (T)10 | 10 | 76122 | 76131 |
| 56 | Mononucleotide | (T)12 | 12 | 76138 | 76149 |
| 57 | Mononucleotide | (A)10 | 10 | 78535 | 78544 |
| 58 | Tetranucleotide | (ATTT)3 | 12 | 79516 | 79527 |
| 59 | Trinucleotide | (TTC)3 | 9 | 79916 | 79924 |
| 60 | Trinucleotide | (TTC)3 | 9 | 80641 | 80649 |
| 61 | Trinucleotide | (AGA)3 | 9 | 86321 | 86329 |
| 62 | Trinucleotide | (AGA)3 | 9 | 87795 | 87803 |
| 63 | Trinucleotide | (AAG)3 | 9 | 90820 | 90828 |
| 64 | Trinucleotide | (AAC)3 | 9 | 91614 | 91622 |
| 65 | Tetranucleotide | (AACG)3 | 12 | 98318 | 98329 |
| 66 | Trinucleotide | (CCT)3 | 9 | 101598 | 101606 |
| 67 | Trinucleotide | (TTG)3 | 9 | 102265 | 102273 |
| 68 | Trinucleotide | (TAA)3 | 9 | 103181 | 103189 |
| 69 | Mononucleotide | (A)10 | 10 | 103959 | 103968 |
| 70 | Tetranucleotide | (AACA)3 | 12 | 104687 | 104698 |
| 71 | Tetranucleotide | (ATTA)5 | 20 | 104773 | 104792 |
| 72 | Tetranucleotide | (AATA)3 | 12 | 106828 | 106839 |
| 73 | Trinucleotide | (TTA)3 | 9 | 112031 | 112039 |
| 74 | Trinucleotide | (AGC)3 | 9 | 112414 | 112422 |
| 75 | Tetranucleotide | (TCGT)3 | 12 | 117015 | 117026 |
| 76 | Trinucleotide | (GTT)3 | 9 | 123723 | 123731 |
| 77 | Trinucleotide | (CTT)3 | 9 | 124517 | 124525 |
| 78 | Trinucleotide | (TTC)3 | 9 | 127541 | 127549 |
| 79 | Trinucleotide | (TCT)3 | 9 | 129016 | 129024 |
| 80 | Trinucleotide | (GAA)3 | 9 | 134696 | 134704 |
