# Supplementary material for: Dig up tall fescue plastid genomes for the identification of morphotype-specific DNA variants
Source: BMC Genomics. 2023 Oct 3;24:586. doi: 10.1186/s12864-023-09631-8 (PMC10546690; doi:10.1186/s12864-023-09631-8)
Supplement: Supplementary file 1 — Additional file 1: Tables S1-S13 [file 12864_2023_9631_MOESM1_ESM.zip › Additional file 1 Table S9_updated_ESM.docx]

**Additional file 1: Table S9**. List of simple sequence repeats (SSRs) identified in Mediterranean cv. Resolute tall fescue plastid genome.

| **SSR number** | **SSR type** | **SSR** | **Size** | **Start (bp)** | **End (bp)** |
| --- | --- | --- | --- | --- | --- |
| 1 | Trinucleotide | (AAG)3 | 9 | 326 | 334 |
| 2 | Trinucleotide | (AAT)3 | 9 | 3550 | 3558 |
| 3 | Mononucleotide | (A)10 | 10 | 3579 | 3588 |
| 4 | Mononucleotide | (A)10 | 10 | 3774 | 3783 |
| 5 | Trinucleotide | (TTA)3 | 9 | 5887 | 5895 |
| 6 | Mononucleotide | (A)10 | 10 | 7346 | 7355 |
| 7 | Mononucleotide | (T)10 | 10 | 7550 | 7559 |
| 8 | Mononucleotide | (T)10 | 10 | 7812 | 7821 |
| 9 | Mononucleotide | (A)11 | 11 | 8022 | 8032 |
| 10 | Mononucleotide | (T)11 | 11 | 8356 | 8366 |
| 11 | Mononucleotide | (A)10 | 10 | 12004 | 12013 |
| 12 | Pentanucleotide | (AGTTT)3 | 15 | 12333 | 12347 |
| 13 | Tetranucleotide | (ATAC)3 | 12 | 16230 | 16241 |
| 14 | Mononucleotide | (A)10 | 10 | 18154 | 18163 |
| 15 | Mononucleotide | (A)13 | 13 | 20625 | 20637 |
| 16 | Trinucleotide | (AAC)3 | 9 | 23399 | 23407 |
| 17 | Mononucleotide | (A)10 | 10 | 24178 | 24187 |
| 18 | Trinucleotide | (TAT)3 | 9 | 24982 | 24990 |
| 19 | Trinucleotide | (AGA)3 | 9 | 26583 | 26591 |
| 20 | Trinucleotide | (AAG)3 | 9 | 27413 | 27421 |
| 21 | Trinucleotide | (ATT)3 | 9 | 29315 | 29323 |
| 22 | Mononucleotide | (A)12 | 12 | 29404 | 29415 |
| 23 | Trinucleotide | (GTT)4 | 12 | 31006 | 31017 |
| 24 | Trinucleotide | (TGC)3 | 9 | 31729 | 31737 |
| 25 | Trinucleotide | (TCT)3 | 9 | 36271 | 36279 |
| 26 | Trinucleotide | (TTG)3 | 9 | 39447 | 39455 |
| 27 | Trinucleotide | (GCA)3 | 9 | 39740 | 39748 |
| 28 | Mononucleotide | (T)10 | 10 | 42219 | 42228 |
| 29 | Trinucleotide | (AGT)3 | 9 | 42304 | 42312 |
| 30 | Tetranucleotide | (AAAC)3 | 12 | 43652 | 43663 |
| 31 | Trinucleotide | (CAA)3 | 9 | 43846 | 43854 |
| 32 | Mononucleotide | (A)12 | 12 | 45428 | 45439 |
| 33 | Trinucleotide | (AAC)3 | 9 | 49122 | 49130 |
| 34 | Mononucleotide | (T)12 | 12 | 49282 | 49293 |
| 35 | Trinucleotide | (TCC)3 | 9 | 52221 | 52229 |
| 36 | Trinucleotide | (AAC)3 | 9 | 53996 | 54004 |
| 37 | Mononucleotide | (A)14 | 14 | 56224 | 56237 |
| 38 | Mononucleotide | (T)10 | 10 | 57870 | 57879 |
| 39 | Trinucleotide | (GAA)3 | 9 | 57906 | 57914 |
| 40 | Mononucleotide | (T)10 | 10 | 58689 | 58698 |
| 41 | Trinucleotide | (TTC)3 | 9 | 60108 | 60116 |
| 42 | Mononucleotide | (G)11 | 11 | 62581 | 62591 |
| 43 | Mononucleotide | (T)10 | 10 | 62744 | 62753 |
| 44 | Mononucleotide | (A)11 | 11 | 63014 | 63024 |
| 45 | Tetranucleotide | (TTCA)3 | 12 | 63621 | 63632 |
| 46 | Trinucleotide | (TTC)4 | 12 | 64659 | 64670 |
| 47 | Mononucleotide | (T)10 | 10 | 65204 | 65213 |
| 48 | Trinucleotide | (AAC)3 | 9 | 65495 | 65503 |
| 49 | Mononucleotide | (T)11 | 11 | 66291 | 66301 |
| 50 | Tetranucleotide | (AGAA)3 | 12 | 67913 | 67924 |
| 51 | Mononucleotide | (A)11 | 11 | 70969 | 70979 |
| 52 | Trinucleotide | (GAT)3 | 9 | 73053 | 73061 |
| 53 | Trinucleotide | (AAG)3 | 9 | 73776 | 73784 |
| 54 | Trinucleotide | (TAT)3 | 9 | 74841 | 74849 |
| 55 | Mononucleotide | (T)10 | 10 | 76046 | 76055 |
| 56 | Mononucleotide | (T)10 | 10 | 76064 | 76073 |
| 57 | Mononucleotide | (T)12 | 12 | 76625 | 76636 |
| 58 | Mononucleotide | (T)10 | 10 | 77175 | 77184 |
| 59 | Mononucleotide | (T)10 | 10 | 79407 | 79416 |
| 60 | Tetranucleotide | (ATTT)3 | 12 | 79458 | 79469 |
| 61 | Trinucleotide | (TTC)3 | 9 | 79858 | 79866 |
| 62 | Trinucleotide | (TTC)3 | 9 | 80589 | 80597 |
| 63 | Mononucleotide | (A)10 | 10 | 83264 | 83273 |
| 64 | Trinucleotide | (AGA)3 | 9 | 86311 | 86319 |
| 65 | Trinucleotide | (AGA)3 | 9 | 87785 | 87793 |
| 66 | Trinucleotide | (AAG)3 | 9 | 90810 | 90818 |
| 67 | Trinucleotide | (AAC)3 | 9 | 91604 | 91612 |
| 68 | Trinucleotide | (GGT)3 | 9 | 95052 | 95060 |
| 69 | Tetranucleotide | (AACG)3 | 12 | 98308 | 98319 |
| 70 | Trinucleotide | (CCT)3 | 9 | 101608 | 101616 |
| 71 | Trinucleotide | (TTG)3 | 9 | 102275 | 102283 |
| 72 | Trinucleotide | (TAA)3 | 9 | 103191 | 103199 |
| 73 | Tetranucleotide | (AACA)3 | 12 | 104646 | 104657 |
| 74 | Tetranucleotide | (ATTA)5 | 20 | 104732 | 104751 |
| 75 | Tetranucleotide | (AATA)3 | 12 | 106787 | 106798 |
| 76 | Trinucleotide | (TTA)3 | 9 | 112020 | 112028 |
| 77 | Trinucleotide | (AGC)3 | 9 | 112403 | 112411 |
| 78 | Tetranucleotide | (TCGT)3 | 12 | 117002 | 117013 |
| 79 | Trinucleotide | (GTT)3 | 9 | 123710 | 123718 |
| 80 | Trinucleotide | (CTT)3 | 9 | 124504 | 124512 |
| 81 | Trinucleotide | (TTC)3 | 9 | 127528 | 127536 |
| 82 | Trinucleotide | (TCT)3 | 9 | 129003 | 129011 |
| 83 | Mononucleotide | (T)10 | 10 | 132002 | 132011 |
| 84 | Trinucleotide | (GAA)3 | 9 | 134678 | 134686 |
