# Supplementary material for: Dig up tall fescue plastid genomes for the identification of morphotype-specific DNA variants
Source: BMC Genomics. 2023 Oct 3;24:586. doi: 10.1186/s12864-023-09631-8 (PMC10546690; doi:10.1186/s12864-023-09631-8)
Supplement: Supplementary file 1 — Additional file 1: Tables S1-S13 [file 12864_2023_9631_MOESM1_ESM.zip › Additional file 1 - Table S13.docx]

**Additional file 1: Table S13**. List of chloroplast genomes used for the genomic divergence analysis in this study.

| Species names | Subfamily^*^ | Chloroplast genome size (bp) | NCBI reference sequence accession number | Date of access to the NCBI GenBank |
| --- | --- | --- | --- | --- |
| Rice (*Oryza sativa*) | Oryzoideae | 134,551 | LC739565.1 | June 14, 2023 |
| Maize (*Zea mays*) | Panicoideae | 140,384 | NC_001666.2 | June 14, 2023 |
| Sorghum (*Sorghum bicolor*) | Panicoideae | 140,754 | NC_008602.1 | June 14, 2023 |
| Perennial ryegrass (*Lolium perenne*) | Pooideae | 135,282 | NC_009950.1 | June 14, 2023 |
| Timothy grass (Phleum pratense) | Pooideae | 134,973 | NC_067044.1 | June 14, 2023 |
| Meadow fescue (*Festuca pratensis*) | Pooideae | 135,291 | NC_019650.1 | June 14, 2023 |
| Sheep fescue (*Festuca ovina*) | Pooideae | 133,165 | NC_019649.1 | June 14, 2023 |
| Kentucky bluegrass (*Poa pratensis*) | Pooideae | 135,649 | NC_057962.1 | June 14, 2023 |
| Barley (*Hordeum vulgare*) | Pooideae | 136,485 | NC_056985.1 | June 14, 2023 |
| Wheat (*Triticum aestivum*) | Pooideae | 134,545 | NC_002762.1 | June 14, 2023 |
| Continental cv. Texoma MaxQ II (*Festuca arundinacea* Schreb.) | Pooideae | 135,283 | OQ928086 | This study |
| Rhizomatous cv. Torpedo (*Festuca arundinacea* Schreb.) | Pooideae | 135,336 | OQ935427 | This study |
| Mediterranean cv. Resolute (*Festuca arundinacea* Schreb.) | Pooideae | 135,324 | OQ935426 | This study |

^*^The subfamily Oryzoideae, Panicoideae, and Pooideae are all belong to the Poaceae family.
