# Supplementary material for: Dig up tall fescue plastid genomes for the identification of morphotype-specific DNA variants
Source: BMC Genomics. 2023 Oct 3;24:586. doi: 10.1186/s12864-023-09631-8 (PMC10546690; doi:10.1186/s12864-023-09631-8)
Supplement: Supplementary file 2 — Additional file 2: Figs. S1-S3 [file 12864_2023_9631_MOESM2_ESM.zip › Additional file 2 Figure S2_updated_ESM.pdf]

(A)

```
                                    20                   40                   60
                                     |                    |                    |
TexomaMaxQII_ccsA  ATGCTATTTG CAACTTTAGA ACATATATTA AATCATATCT CCTTCTCAAC CATTTCCATT 60
     Torpedo_ccsA  ATGCTATTTG CAACTTTAGA ACATATATTA AATCATATCT CCTTCTCAAC CATTTCCATT 60
    Resolute_ccsA  ATGCTATTTG CAACTTTAGA ACATATATTA AATCATATCT CCTTCTCAAC CATTTCCATT 60
                                    80                  100                  120
                                     |                    |                    |
TexomaMaxQII_ccsA  GTGATTACGA TTCATTTGAT AACCTTATTA GTTCATGAAC TTGGGGGATT ACGTGATTCG 120
     Torpedo_ccsA  GTGATTACGA TTCATTTGAT AACCTTATTA GTTCATGAAC TTGGGGGATT ACGTGATTCG 120
    Resolute_ccsA  GTGATTACGA TTCATTTGAT AACCTTATTA GTTCATGAAC TTGGGGGATT ACGTGATTCG 120
                                   140                  160                  180
                                     |                    |                    |
TexomaMaxQII_ccsA  TCAGAAAAAG GAATGATAGT TACTTTTTTC TCTATAACAG GATTCCTAGT TTCTCGCTGG 180
     Torpedo_ccsA  TCAGAAAAAG GAATGATAGT TACTTTTTTC TCTATAACAG GATTCCTAGT TTCTCGCTGG 180
    Resolute_ccsA  TCAGAAAAAG GAATGATAGT TACTTTTTTC TCTATAACAG GATTCCTAGT TTCTCGCTGG 180
                                   200                  220                  240
                                     |                    |                    |
TexomaMaxQII_ccsA  GCTTCTTCGG GACATTTTCC ATTAAGTAAT TTATATGAGT CGTTGATCTT CCTTTCATGG 240
     Torpedo_ccsA  GCTTCTTCGG GACATTTTCC ATTAAGTAAT TTATATGAGT CGTTGATCTT CCTTTCATGG 240
    Resolute_ccsA  GCTTCTTCGG GACATTTTCC ATTAAGTAAT TTATATGAGT CGTTGATCTT CCTTTCATGG 240
                                   260                  280                  300
                                     |                    |                    |
TexomaMaxQII_ccsA  GCTCTGTATA TTCTTCATAC CATTCCTAAG ATACAGAACT CTAAAAATGA TTTAAGCACA 300
     Torpedo_ccsA  GCTCTGTATA TTCTTCATAC CATTCCTAAG ATACAGAACT CTAAAAATGA TTTAAGCACA 300
    Resolute_ccsA  GCTCTGTATA TTCTTCATAC CATTCCTAAG ATACAGAACT CTAAAAATGA TTTAAGCACA 300
                                   320                  340                  360
                                     |                    |                    |
TexomaMaxQII_ccsA  ATAACTACGC CAAGTACTAT TTTAACGCAA GGCTTTGCCA CATCGGGTCT TTTAACTGAA 360
     Torpedo_ccsA  ATAACTACGC CAAGTACTAT TTTAACGCAA GGCTTTGCCA CATCGGGTCT TTTAACTGAA 360
    Resolute_ccsA  ATAACTACGC CAAGTACTAT TTTAACGCAA GGCTTTGCCA CATCGGGTCT TTTAACTGAA 360
                                   380                  400                  420
                                     |                    |                    |
TexomaMaxQII_ccsA  ATGCATCAAT CCACAATACT AGTACCCGCT CTCCAATCTC AGTGGTTAAT GATGCATGTC 420
     Torpedo_ccsA  ATGCATCAAT CCACAATACT AGTACCCGCT CTCCAATCTC AGTGGTTAAT GATGCATGTC 420
    Resolute_ccsA  ATGCATCAAT CCACAATACT AGTACCCGCT CTCCAATCTC AGTGGTTAAT GATGCATGTC 420
                                   440                  460                  480
                                     |                    |                    |
TexomaMaxQII_ccsA  AGTATGATGT TACTAAGCTA TGCAACTCTT TTGTGCGGAT CCTTATTATC TGCCGCTATT 480
     Torpedo_ccsA  AGTATGATGT TACTAAGCTA TGCAACTCTT TTGTGCGGAT CCTTATTATC TGCCGCTATT 480
    Resolute_ccsA  AGTATGATGT TACTAAGCTA TGCAACTCTT TTGTGCGGAT CTTTATTATC TGCCGCTATT 480
                                   500                  520                  540
                                     |                    |                    |
TexomaMaxQII_ccsA  CTAATCATTA GATTTCGAAA TAATTTCTTT TTCTTTTCTA AAAAGAAAAA ------AAAT 534
     Torpedo_ccsA  CTAATCATTA GATTTCGAAA TAATTTCTTT TTCTTTTCTA AAAAGAAAAA ------AAAT 534
    Resolute_ccsA  CTAATCATTA GATTTCGAAA TAATTTCTTT TTCTTTTCTA AAAAGAAAAA GAAAAAAAAT 540
                                   560                  580                  600
                                     |                    |                    |
TexomaMaxQII_ccsA  GTTTTACTTA AAACATTTTT CTTTAGTGAT TTCTATGTAA AAAGAAGTTC TTTAAAAAGC 594
     Torpedo_ccsA  GTTTTACTTA AAACATTTTT CTTTAGTGAT TTCTATGTAA AAAGAAGTTC TTTAAAAAGC 594
    Resolute_ccsA  GTTTTACTTA AAACATTTTT CTTTAGTGAT TTCTATGTAA AAAGAAGTTC TTTAAAAAGC 600
                                   620                  640                  660
                                     |                    |                    |
TexomaMaxQII_ccsA  ACCTCTGTTC CTTCATTCCC AAATTATTAC AAATATCAAT TAACGGAGCG TTTAGATTCT 654
     Torpedo_ccsA  ACCTCTGTTC CTTCATTCCC AAATTATTAC AAATATCAAT TAACGGAGCG TTTAGATTCT 654
    Resolute_ccsA  ACCTCTGTTC CTTCATTCCC AAATTATTAC AAATATCAAT TAACGGAGCG TTTAGATTCT 660
                                   680                  700                  720
                                     |                    |                    |
TexomaMaxQII_ccsA  TGGAGTTATC GTGTCATTAG CCTAGGATTT ACCCTTTTAA CCATAGGTAT TCTTTGTGGA 714
     Torpedo_ccsA  TGGAGTTATC GTGTCATTAG CCTAGGATTT ACCCTTTTAA CCATAGGTAT TCTTTGTGGA 714
    Resolute_ccsA  TGGAGTTATC GTGTCATTAG CCTAGGATTT ACCCTTTTAA CCATAGGTAT TCTTTGTGGA 720
                                   740                  760                  780
                                     |                    |                    |
TexomaMaxQII_ccsA  GCAGTATGGG CTAATGAGGC GTGGGGATCC TATTGGAATT GGGATCCTAA GGAAACTTGG 774
     Torpedo_ccsA  GCAGTATGGG CTAATGAGGC GTGGGGATCC TATTGGAATT GGGATCCTAA GGAAACTTGG 774
    Resolute_ccsA  GCAGTATGGG CTAATGAGGC GTGGGGATCC TATTGGAATT GGGATCCTAA GGAAACTTGG 780
                                   800                  820                  840
                                     |                    |                    |
TexomaMaxQII_ccsA  GCATTTATTA CTTGGACCAT ATTTGCAATT TATTTACATA GTAGAACAAA TCTAAATTGG 834
     Torpedo_ccsA  GCATTTATTA CTTGGACCAT ATTTGCAATT TATTTACATA GTAGAACAAA TCTAAATTGG 834
    Resolute_ccsA  GCATTTATTA CTTGGACCAT ATTTGCAATT TATTTACATA GTAGAACAAA TCTAAATTGG 840
                                   860                  880                  900
                                     |                    |                    |
TexomaMaxQII_ccsA  AAGGGTACGA ATTCCGCACT TGTAGCTTCG ATAGGATTTC TTATAATTTG GATCTGCTAT 894
     Torpedo_ccsA  AAGGGTACGA ATTCCGCACT TGTAGCTTCG ATAGGATTTC TTATAATTTG GATCTGCTAT 894
    Resolute_ccsA  AAGGGTACGA ATTCTGCACT TGTAGCTTCG ATAGGATTTC TTATAATTTG GATCTGCTAT 900
                                   920                  940                  960
                                     |                    |                    |
TexomaMaxQII_ccsA  TTTGGTATCA ATCTATTAGG AATAGGTTTA CATAGTTATG GTTCGTTTAT ATTAACATCT 954
     Torpedo_ccsA  TTTGGTATCA ATCTATTAGG AATAGGTTTA CATAGTTATG GTTCGTTTAT ATTAACATCT 954
    Resolute_ccsA  TTTGGTATCA ATCTATTAGG AATAGGTTTA CATAGTTATG GTTCGTTTAT ATTAACACCT 960

TexomaMaxQII_ccsA  AAATGA 960
     Torpedo_ccsA  AAATGA 960
    Resolute_ccsA  AAATGA 966
```

(B)

```
                                      20                                      40                                      60
                                       |                                       |                                       |
TexomaMaxQII_ccsA  MLFATLEHILNHISFSTISIVITIHLITLLVHELGGLRDSSEKGMIVTFFSITGFLVSRW  60
     Torpedo_ccsA  MLFATLEHILNHISFSTISIVITIHLITLLVHELGGLRDSSEKGMIVTFFSITGFLVSRW  60
   Resolute_ccsA  MLFATLEHILNHISFSTISIVITIHLITLLVHELGGLRDSSEKGMIVTFFSITGFLVSRW  60

                                      80                                     100                                     120
                                       |                                       |                                       |
TexomaMaxQII_ccsA  ASSGHFPLSNLYESLIFLSWALYILHTIPKIQNSKNDLSTITTPSTILTQGFATSGLLTE 120
     Torpedo_ccsA  ASSGHFPLSNLYESLIFLSWALYILHTIPKIQNSKNDLSTITTPSTILTQGFATSGLLTE 120
   Resolute_ccsA  ASSGHFPLSNLYESLIFLSWALYILHTIPKIQNSKNDLSTITTPSTILTQGFATSGLLTE 120

                                     140                                     160                                     180
                                       |                                       |                                       |
TexomaMaxQII_ccsA  MHQSTILVPALQSQWLMMHVSMMLLSYATLLCGSLLSAAILIIRFRNNFFFFSKKKK--N 178
     Torpedo_ccsA  MHQSTILVPALQSQWLMMHVSMMLLSYATLLCGSLLSAAILIIRFRNNFFFFSKKKK--N 178
   Resolute_ccsA  MHQSTILVPALQSQWLMMHVSMMLLSYATLLCGSLLSAAILIIRFRNNFFFFSKKKKKKN 180

                                     200                                     220                                     240
                                       |                                       |                                       |
TexomaMaxQII_ccsA  VLLKTFFFSDFYVKRSSLKSTSVPSFPNYYKYQLTERLDSWSYRVISLGFTLLTIGILCG 238
     Torpedo_ccsA  VLLKTFFFSDFYVKRSSLKSTSVPSFPNYYKYQLTERLDSWSYRVISLGFTLLTIGILCG 238
   Resolute_ccsA  VLLKTFFFSDFYVKRSSLKSTSVPSFPNYYKYQLTERLDSWSYRVISLGFTLLTIGILCG 240

                                     260                                     280                                     300
                                       |                                       |                                       |
TexomaMaxQII_ccsA  AVWANEAWGSYWNWDPKETWAFITWTIFAIYLHSRTNLNWKGTNSALVASIGFLIIWICY 298
     Torpedo_ccsA  AVWANEAWGSYWNWDPKETWAFITWTIFAIYLHSRTNLNWKGTNSALVASIGFLIIWICY 298
   Resolute_ccsA  AVWANEAWGSYWNWDPKETWAFITWTIFAIYLHSRTNLNWKGTNSALVASIGFLIIWICY 300

                                     320
                                       |
TexomaMaxQII_ccsA  FGINLLGIGLHSYGSFILTSK 319
     Torpedo_ccsA  FGINLLGIGLHSYGSFILTSK 319
   Resolute_ccsA  FGINLLGIGLHSYGSFILTPK 321
```

(C)

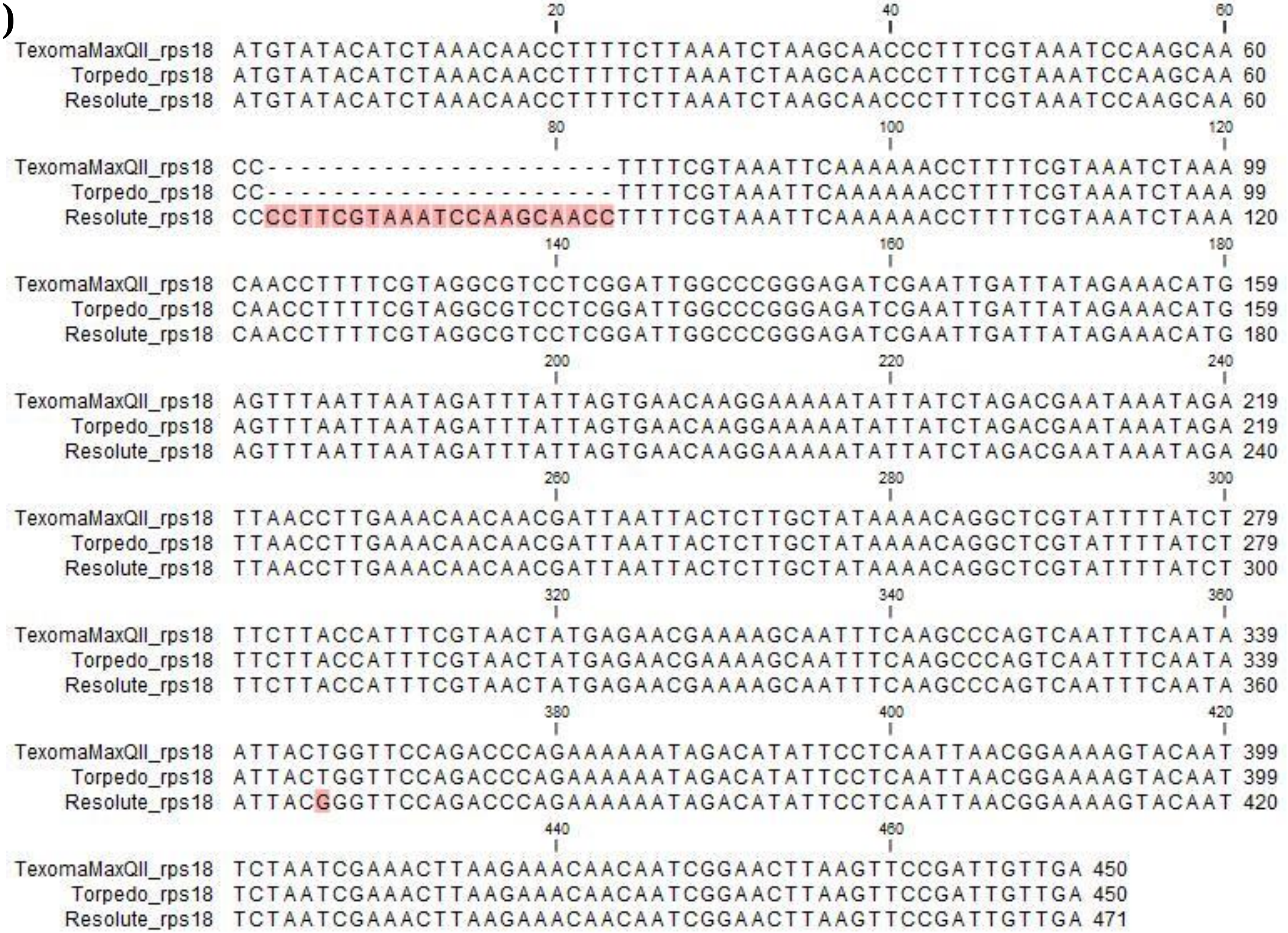

20 40 60
TexomaMaxQII_rps18 ATGTATACATCTAAACAACCTTTTCTTAAATCTAAGCAACCCTTTCGTAAATCCAAGCAA 60
Torpedo_rps18 ATGTATACATCTAAACAACCTTTTCTTAAATCTAAGCAACCCTTTCGTAAATCCAAGCAA 60
Resolute_rps18 ATGTATACATCTAAACAACCTTTTCTTAAATCTAAGCAACCCTTTCGTAAATCCAAGCAA 60
80 100 120
TexomaMaxQII_rps18 CC---------------------TTTTCGTAAATTCAAAAAACCTTTTCGTAAATCTAAA 99
Torpedo_rps18 CC---------------------TTTTCGTAAATTCAAAAAACCTTTTCGTAAATCTAAA 99
Resolute_rps18 CCCCTTCGTAAATCCAAGCAACCTTTTCGTAAATTCAAAAAACCTTTTCGTAAATCTAAA 120
140 160 180
TexomaMaxQII_rps18 CAACCTTTTCGTAGGCGTCCTCGGATTGGCCCGGGAGATCGAATTGATTATAGAAACATG 159
Torpedo_rps18 CAACCTTTTCGTAGGCGTCCTCGGATTGGCCCGGGAGATCGAATTGATTATAGAAACATG 159
Resolute_rps18 CAACCTTTTCGTAGGCGTCCTCGGATTGGCCCGGGAGATCGAATTGATTATAGAAACATG 180
200 220 240
TexomaMaxQII_rps18 AGTTTAATTAATAGATTTATTAGTGAACAAGGAAAAATATTATCTAGACGAATAAATAGA 219
Torpedo_rps18 AGTTTAATTAATAGATTTATTAGTGAACAAGGAAAAATATTATCTAGACGAATAAATAGA 219
Resolute_rps18 AGTTTAATTAATAGATTTATTAGTGAACAAGGAAAAATATTATCTAGACGAATAAATAGA 240
260 280 300
TexomaMaxQII_rps18 TTAACCTTGAAACAACAACGATTAATTACTCTTGCTATAAAACAGGCTCGTATTTTATCT 279
Torpedo_rps18 TTAACCTTGAAACAACAACGATTAATTACTCTTGCTATAAAACAGGCTCGTATTTTATCT 279
Resolute_rps18 TTAACCTTGAAACAACAACGATTAATTACTCTTGCTATAAAACAGGCTCGTATTTTATCT 300
320 340 360
TexomaMaxQII_rps18 TTCTTACCATTTCGTAACTATGAGAACGAAAAGCAATTTCAAGCCCAGTCAATTTCAATA 339
Torpedo_rps18 TTCTTACCATTTCGTAACTATGAGAACGAAAAGCAATTTCAAGCCCAGTCAATTTCAATA 339
Resolute_rps18 TTCTTACCATTTCGTAACTATGAGAACGAAAAGCAATTTCAAGCCCAGTCAATTTCAATA 360
380 400 420
TexomaMaxQII_rps18 ATTACTGGTTCCAGACCCAGAAAAAATAGACATATTCCTCAATTAACGGAAAAGTACAAT 399
Torpedo_rps18 ATTACTGGTTCCAGACCCAGAAAAAATAGACATATTCCTCAATTAACGGAAAAGTACAAT 399
Resolute_rps18 ATTACGGGTTCCAGACCCAGAAAAAATAGACATATTCCTCAATTAACGGAAAAGTACAAT 420
440 460
TexomaMaxQII_rps18 TCTAATCGAAACTTAAGAAACAACAATCGGAACTTAAGTTCCGATTGTTGA 450
Torpedo_rps18 TCTAATCGAAACTTAAGAAACAACAATCGGAACTTAAGTTCCGATTGTTGA 450
Resolute_rps18 TCTAATCGAAACTTAAGAAACAACAATCGGAACTTAAGTTCCGATTGTTGA 471

(D)

```
                                           20                  40                  60
                                           |                   |                   |
TexomaMaxQII_rps18   MYTSKQPFLKSKQPFRKSKQP-------FRKFKKPFRKSKQPFRRRPRIGPGDRIDYRNM  53
     Resolute_rps18  MYTSKQPFLKSKQPFRKSKQPLRKSKQPFRKFKKPFRKSKQPFRRRPRIGPGDRIDYRNM  60
      Torpedo_rps18  MYTSKQPFLKSKQPFRKSKQP-------FRKFKKPFRKSKQPFRRRPRIGPGDRIDYRNM  53

                                           80                  100                 120
                                           |                   |                   |
TexomaMaxQII_rps18   SLINRFISEQGKILSRRINRLTLKQQRLITLAIKQARILSFLPFRNYENEKQFQAQSISI  113
     Resolute_rps18  SLINRFISEQGKILSRRINRLTLKQQRLITLAIKQARILSFLPFRNYENEKQFQAQSISI  120
      Torpedo_rps18  SLINRFISEQGKILSRRINRLTLKQQRLITLAIKQARILSFLPFRNYENEKQFQAQSISI  113

                                           140
                                           |
TexomaMaxQII_rps18   ITGSRPRKNRHIPQLTEKYNSNRNLRNNNRNLSSDC  149
     Resolute_rps18  ITGSRPRKNRHIPQLTEKYNSNRNLRNNNRNLSSDC  156
      Torpedo_rps18  ITGSRPRKNRHIPQLTEKYNSNRNLRNNNRNLSSDC  149
```

(E)

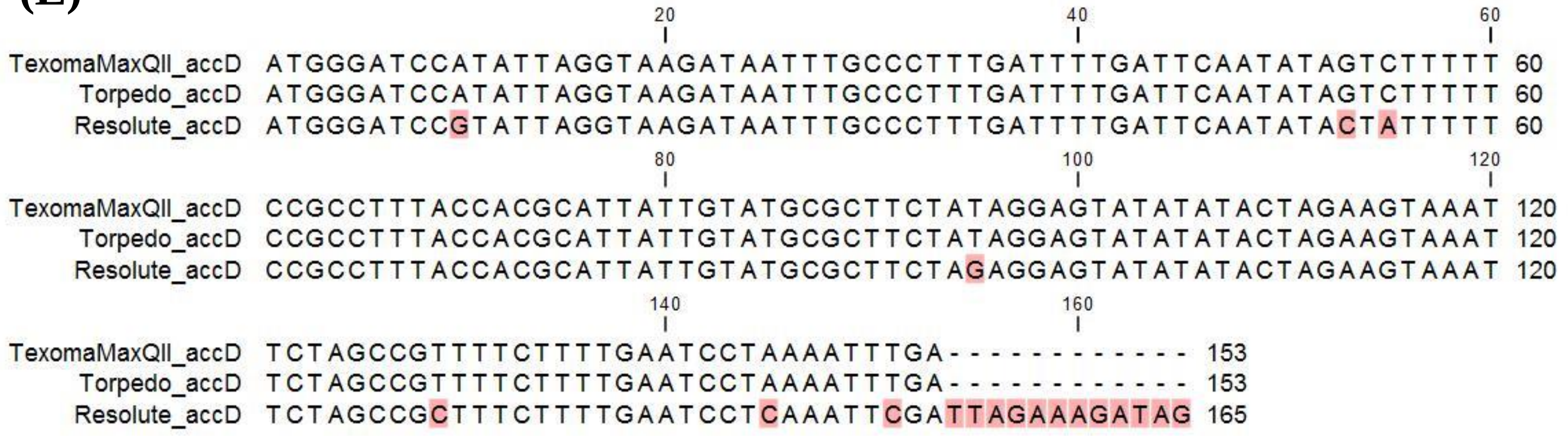
20
40
60
TexomaMaxQII_accD ATGGGATCCATATTAGGTAAGATAATTTGCCCTTTGATTTTGATTCAATATAGTCTTTTT 60
Torpedo_accD ATGGGATCCATATTAGGTAAGATAATTTGCCCTTTGATTTTGATTCAATATAGTCTTTTT 60
Resolute_accD ATGGGATCCGTATTAGGTAAGATAATTTGCCCTTTGATTTTGATTCAATATACTATTTTT 60
80
100
120
TexomaMaxQII_accD CCGCCTTTACCACGCATTATTGTATGCGCTTCTATAGGAGTATATATACTAGAAGTAAAT 120
Torpedo_accD CCGCCTTTACCACGCATTATTGTATGCGCTTCTATAGGAGTATATATACTAGAAGTAAAT 120
Resolute_accD CCGCCTTTACCACGCATTATTGTATGCGCTTCTAGAGGAGTATATATACTAGAAGTAAAT 120
140
160
TexomaMaxQII_accD TCTAGCCGTTTTCTTTTGAATCCTAAAATTTGA------------ 153
Torpedo_accD TCTAGCCGTTTTCTTTTGAATCCTAAAATTTGA------------ 153
Resolute_accD TCTAGCCGCTTTCTTTTGAATCCTCAAATTCGATTAGAAAGATAG 165

(F)

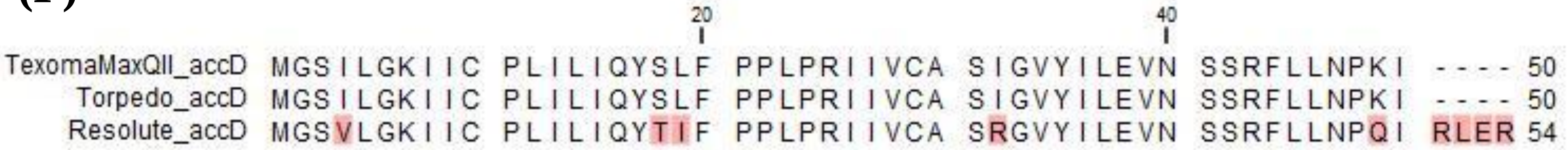
20
40
TexomaMaxQII_accD MGSILGKIIC PLILIQYSLF PPLPRIIVCA SIGVYILEVN SSRFLLNPKI ---- 50
Torpedo_accD MGSILGKIIC PLILIQYSLF PPLPRIIVCA SIGVYILEVN SSRFLLNPKI ---- 50
Resolute_accD MGSVLGKIIC PLILIQYTIF PPLPRIIVCA SRGVYILEVN SSRFLLNPQI RLER 54

(G)

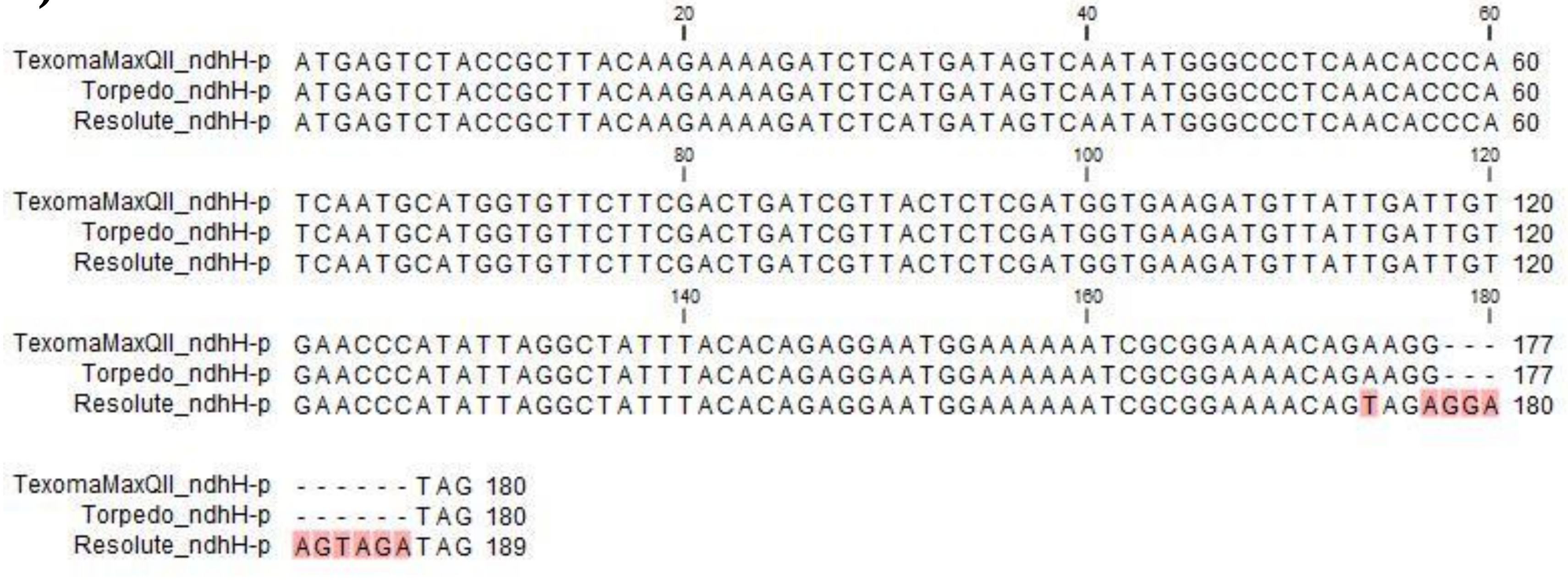

(H)

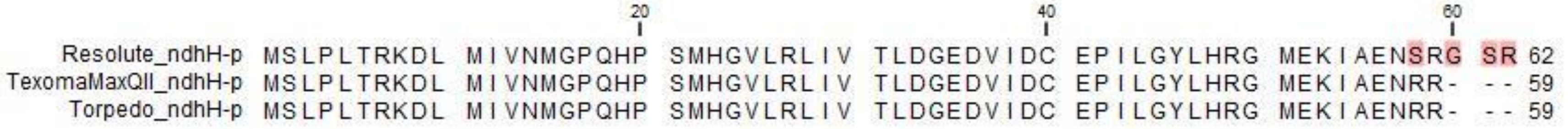

Additional file 2: Figure S2. Alignment of *ccsA* (A and B), *rps12* (C and D), *accD* (E and F), and *ndhH*-p (G and H) gene sequences of three tall fescue morphotypes. Figure A, C, E, and G are nucleotide sequences, and Figure B, D, F, and H are protein sequences.
