# Supplementary figures and images for: Dig up tall fescue plastid genomes for the identification of morphotype-specific DNA variants
Source: BMC Genomics. 2023 Oct 3;24:586. doi: 10.1186/s12864-023-09631-8 (PMC10546690; doi:10.1186/s12864-023-09631-8)

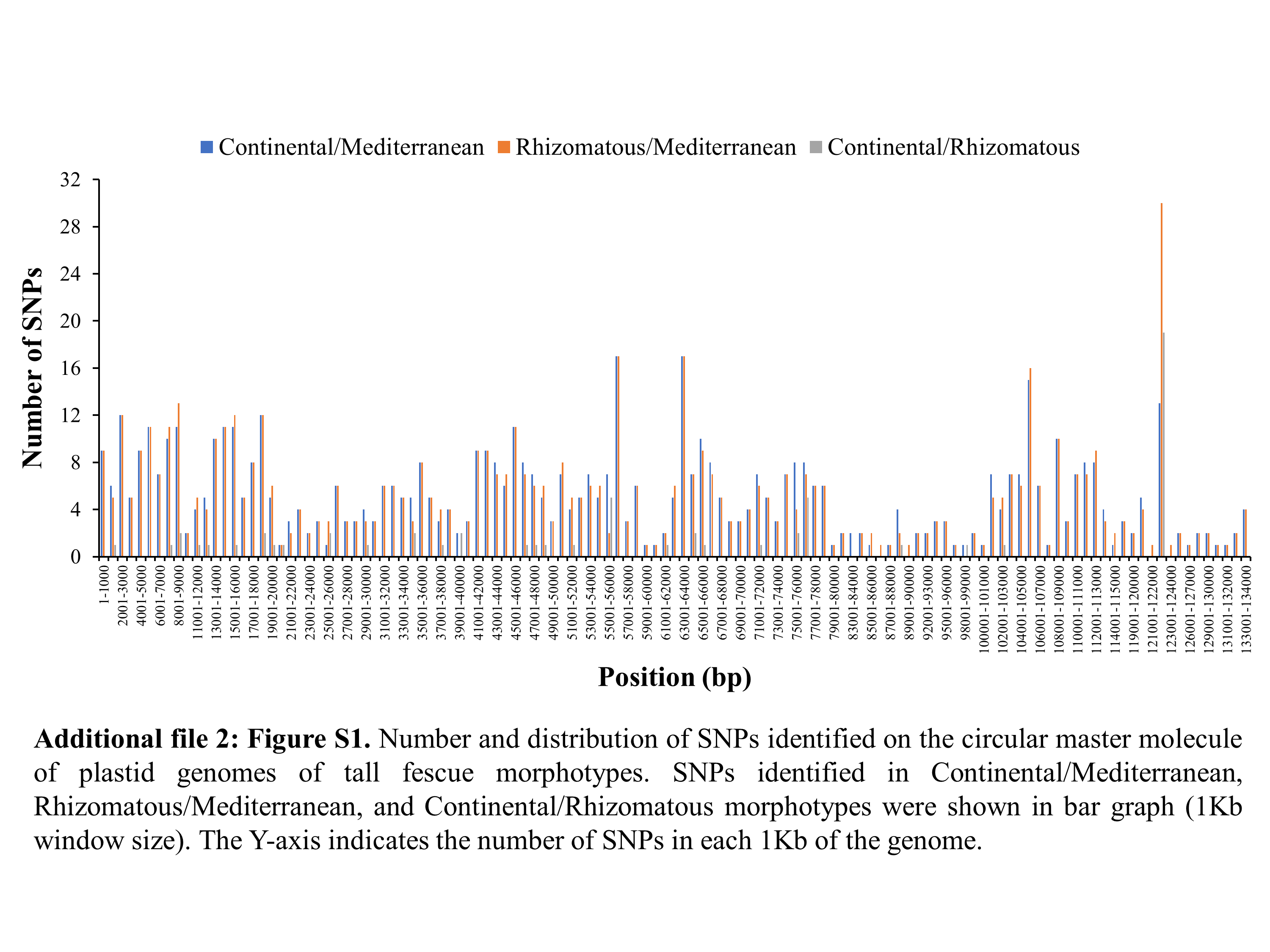

Supplement: Supplementary file 2 — Additional file 2: Figs. S1-S3 [file 12864_2023_9631_MOESM2_ESM.zip › Additional file 2 Figure S1_updated_ESM.tif]

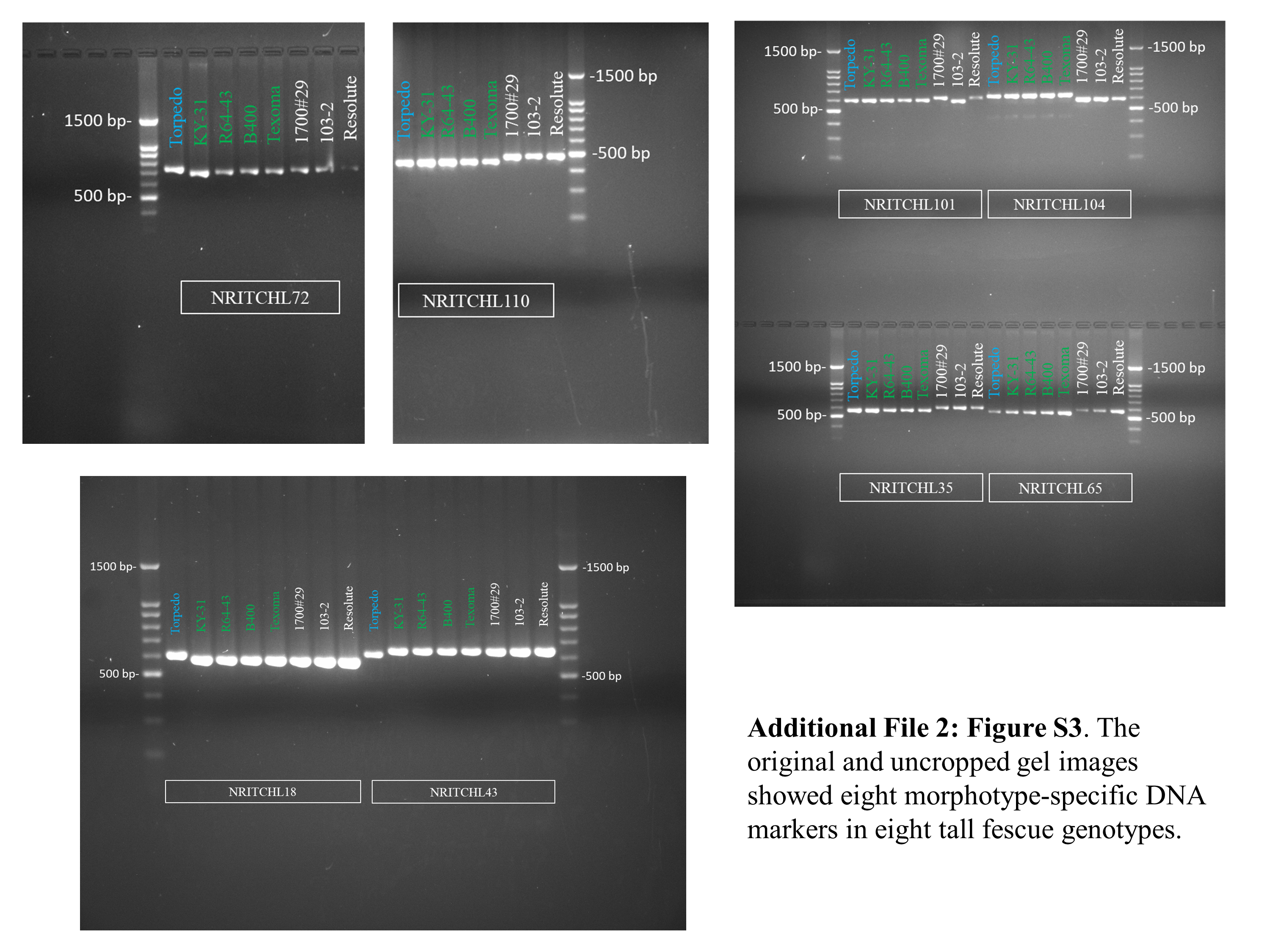

Supplement: Supplementary file 2 — Additional file 2: Figs. S1-S3 [file 12864_2023_9631_MOESM2_ESM.zip › Additional file 2 - Fig. S3.png]
